# Stakeholder Perspectives on National Implementation of Financial Incentives for Smoking Cessation Before, During and After Pregnancy: A Policy Lab Study

**DOI:** 10.1111/hex.70893

**Published:** 2026-10-12

**Authors:** Linda van der Spek, Niek Mouter, Tessa Scheffers‐van Schayck, Leonieke J. Breunis, Hans van Kippersluis, Stijn Hooijman, Jasper V. Been

**Affiliations:** ^1^ Division of Neonatology, Department of Neonatal and Paediatric Intensive Care, Erasmus MC Sophia Children's Hospital University Medical Centre Rotterdam Rotterdam The Netherlands; ^2^ Department of Epidemiology Trimbos Institute Utrecht The Netherlands; ^3^ Department of Engineering Systems and Services, Faculty of Technology Policy and Management Delft University of Technology Delft The Netherlands; ^4^ Department of Tobacco Control Trimbos Institute Utrecht The Netherlands; ^5^ Department of Obstetrics and Gynaecology, Erasmus MC Sophia Children's Hospital University Medical Centre Rotterdam Rotterdam The Netherlands; ^6^ Department of Paediatrics, Erasmus MC Sophia Children's Hospital University Medical Centre Rotterdam Rotterdam The Netherlands; ^7^ Erasmus School of Economics Erasmus University Rotterdam Rotterdam The Netherlands

**Keywords:** financial incentives, implementation, parents, policy lab, pregnancy, smoking cessation, stakeholder perspectives

## Abstract

**Background:**

Financial incentives can substantially increase effectiveness of smoking cessation support for pregnant women, their partners, people planning a pregnancy, and parents, yet implementation in routine care remains limited. This study explored how incentives could be implemented within a national cessation programme through collaboration with key stakeholders.

**Methods:**

A Policy Lab approach was used to collaboratively identify barriers, opportunities, and policy directions for implementation. Two interactive sessions were held in autumn 2024 with representatives of the Dutch Ministry of Health, the National Health Care Institute, health insurers, researchers, healthcare professionals, a smoking cessation support provider, and a parent. Transcripts and exercise outputs were thematically analysed using the Consolidated Framework for Implementation Research (CFIR). As part of the policy lab process, we developed a policy brief, infographic, and explainer video.

**Results:**

Key challenges included limited public and political support, legal and financial barriers, and logistical and design‐related issues. Participants recommended clear messaging on health and societal benefits; initiating pilot projects and providing clear insight into the costs, benefits, and timing of returns; and integrating incentives into existing cessation programme structures with accessible and feasible design.

**Conclusion:**

This study provides practical guidance for policymakers and health insurers to integrate financial incentives into smoking cessation support for (expectant) parents, while offering transferable insights for implementation in other settings.

**Patient or Public Contribution:**

A parent with lived experience of receiving smoking cessation support with incentives participated in the Policy Lab. In addition, healthcare professionals, smoking cessation programme providers, health insurers, and representatives from public bodies contributed perspectives grounded in their roles representing patient and public interests. These inputs informed the development of recommendations and dissemination materials, including the policy brief and explainer video, to enhance relevance, accessibility, and public acceptability.

## 1 Introduction

Tobacco smoking is a leading contributor to health disparities, with smoking during pregnancy representing the primary modifiable risk factor for adverse foetal and child health outcomes, including stillbirth and low birth weight [1, 2]. In the Netherlands, disparities are reflected in 2023 national surveillance data: although 6% of women overall report smoking at some point during pregnancy, this proportion was 20% among women with a lower educational level, compared with only 3% of highly educated women [3] Financial incentives for validated smoking cessation show considerable promise in cost‐effectively promoting cessation, particularly among pregnant women [4, 5]. A recent meta‐analysis found that 13% of women receiving financial incentives achieved long‐term smoking abstinence, compared with 6% in control groups, with follow‐up extending up to 24 weeks postpartum [5] Reflecting this evidence, the UK National Institute for Health and Care Excellence (NICE) recommends the routine use of financial incentives as part of cessation support for pregnant smokers [6].

Despite the compelling evidence, implementation of incentive‐based cessation programmes outside the UK remains limited. Barriers include public concerns that incentives ‘reward’ individuals for quitting a self‐inflicted behaviour. These concerns reduce political and societal support, challenge prioritisation of such programmes, and thereby so far hold off the translation of trial outcomes into sustainable real‐world implementation [5, 7, 8, 9]. While some of these barriers reflect universal challenges, others depend on local health system structures, policy arrangements, cultural norms surrounding prevention and fairness [9] and public acceptability of financial incentives [7].

To support wider adoption, participatory studies are needed to examine how incentive‐based cessation programmes can be designed and implemented in ways that are acceptable, feasible, and sustainable within national contexts [9, 10, 11]. These studies not only generate context‐specific solutions but also enhance understanding of broader implementation dynamics that may be relevant across settings. Policy labs provide such a participatory approach, enabling diverse stakeholders ‐ academics, policymakers, funders, practitioners, and end‐users – to combine scientific evidence with practice‐based and experiential knowledge and collaboratively explore challenges and opportunities for policy innovation [12, 13].

In this study, we established a policy lab to explore pathways for implementation of financial incentives within the Dutch context of smoking cessation support, particularly through *Smoke‐Free Parents* (SFP), a national telephone counselling programme for pregnant women, their partners, people planning a pregnancy, and parents [14, 15]. These groups are collectively referred to as ‘(expectant) parents’ throughout this paper. Through two interactive sessions, the lab identified barriers, facilitators, and concrete policy recommendations. Although grounded in the Dutch context, the findings provide transferable insights for other countries aiming to integrate financial incentives into cessation care.

## 2 Materials and Methods

### 2.1 Design

We applied a policy lab approach following the framework of Hinrichs‐Krapels et al. [13], within the Participatory Health Research paradigm [16, 17, 18]. Reporting followed the Standards for Reporting Qualitative Research (SRQR) [19] A completed SRQR checklist is available in Supporting File 1. To ensure accessible and policy‐relevant dissemination, findings were translated into a policy brief, infographic, and explainer video.

### 2.2 Researcher Characteristics

The study was conducted by an interdisciplinary team with expertise in epidemiology, obstetrics, neonatology, paediatrics, public health, tobacco control, participatory policy design, and health and behavioural economics. The policy lab sessions were facilitated by NM, an experienced researcher in participatory policy design. The team's complementary expertise and reflexive discussions supported interpretive rigour.

### 2.3 Study Context

In the Netherlands, smoking cessation support, including counselling and pharmacotherapy, is covered under the basic health insurance package (*basisverzekering*). All citizens are required by law to have basic health insurance, which is provided by one of several competing private, non‐profit insurers [20] This system is governed by the Health Insurance Act (*Zorgverzekeringswet*), which defines healthcare as services directly related to individual treatment or cure. The National Health Care Institute (*Zorginstituut Nederland*) determines which services qualify for reimbursement based on effectiveness and necessity, while the Dutch Healthcare Authority (*Nederlandse Zorgautoriteit*) monitors accessibility and fair competition. In addition to basic health insurance, insurers may offer supplementary insurance (*aanvullende verzekering*) on a voluntary basis. Under the Public Health Act (*Wet publieke gezondheid*), municipalities are responsible for implementing collective prevention such as vaccination, screening, and health promotion. They may also fund local prevention projects, such as “Smoke‐Free Generation” campaigns [21] or smoking cessation courses, using prevention budgets.

### 2.4 Participants and Recruitment

A purposive sampling strategy was used to recruit approximately ten stakeholder representatives with diverse roles in smoking cessation policy and practice. This sample size was chosen a priori to facilitate rich, interactive discussions and subgroup activities [22] Inclusion criteria required participants to represent one of the following stakeholder groups: (1) policymakers (i.e., representatives of the Dutch Ministry of Health, Welfare and Sport and policy advisors from the National Health Care Institute); (2) potential financers of the intervention (i.e., health insurers); (3) providers of smoking cessation support including the SFP programme; (4) researchers with expertise in tobacco control and financial incentives; (5) healthcare professionals (e.g., midwives); (6) end‐users (i.e., (expectant) parents who currently smoke or have quit with support involving financial incentives); and (7) members of the national Taskforce Smoke‐Free Start [23] or the SFP implementation team. Individuals under the age of 18 were not eligible. Recruitment took place between May and October 2024 through email, LinkedIn, snowball sampling, and direct outreach. Interested individuals received an information sheet and consent form, which they signed before the first session.

Twelve stakeholder representatives (seven women and five men), excluding two facilitators (NM and LvdS), participated in at least one of the two sessions. The first session involved 11 participants: one university researcher, one representative from the Dutch Ministry of Health, one representative with joint affiliation at the Dutch Ministry of Health and a university, two representatives from the National Health Care Institute, two representatives of health insurers, an implementation leader of the SFP programme, a representative of an organisation providing SFP smoking cessation support, a target group representative, and a physician‐researcher. During the second session, five participants attended: a health insurer representative, an SFP implementation leader, a National Health Care Institute representative, and two physician‐researchers. Attendance was lower in the second, in‐person session at Erasmus MC compared with the first online session, mainly due to logistical constraints. The session built on the first meeting and generated rich discussion. To maximise representation of stakeholder perspectives, several absent participants were contacted individually for follow‐up, and all participants later provided written feedback on the draft policy brief to validate the findings. Separate preparatory discussions were also held with the chief economist of the Dutch Healthcare Authority and the chair of the Dutch Partnership Smoking Cessation.

### 2.5 Ethics

The Erasmus MC Non‐WMO Review Committee concluded that this study was not subject to the Dutch *Medical Research Involving Human Subjects Act (WMO)* and approved its rationale, data collection, and processing procedures (MEC‐2024–0309). All procedures complied with the General Data Protection Regulation (GDPR) and the Dutch General Data Protection Regulation Implementation Act (UAVG). Before participation, all invited stakeholders received written information about the study's aims, procedures, and their rights, and provided written informed consent. Audio recordings and transcripts were securely stored and anonymized before analysis. Participants received travel reimbursement and a financial compensation of €50 per session for their time and contribution.

### 2.6 Procedures

#### 2.6.1 Pre‐Session Activities

Participants received a briefing pack (Supporting File 2), to ensure that all participants entered the policy lab with a shared knowledge base and to stimulate early engagement with the topic. It contained scientific evidence on the effectiveness of financial incentives for smoking cessation, findings on public acceptability, user experiences and unintended consequences, and ethical and equity considerations.

In addition, an online baseline questionnaire was distributed 2 weeks before the first session consisting of three Likert‐scale questions with optional open‐text comments to elicit perspectives on the overall perceived effectiveness of financial incentives and the desirability of funding them through either government or health insurers. The baseline questionnaire served to gain insight into participants' initial opinions regarding the use of incentives, to encourage reflection and involvement ahead of the sessions, and to inform the facilitator in tailoring the discussions to potential areas of divergence or concern. Nine of the twelve invited participants completed the questionnaire (75% response rate).

#### 2.6.2 Policy Lab Sessions

Two interactive policy lab sessions of 2 h each were held in October and November 2024. The first session, conducted online via Microsoft Teams, focused on (a) sharing key insights from the briefing pack and (b) identifying implementation challenges and opportunities through a *pre‐mortem* exercise [24] In this exercise, participants were asked to imagine that the implementation of financial incentives for smoking cessation had already failed and to individually identify potential reasons for this failure, followed by a group discussion to prioritise the five most critical risks. This “prospective hindsight” technique is increasingly applied in implementation and policy research to encourage critical reflection, surface implicit barriers, and reduce overconfidence and groupthink by allowing participants to anticipate potential points of failure and develop actionable strategies [25] Using dot voting and group discussion, participants subsequently identified priority areas and feasible solutions to facilitate implementation. A shared online Miro Board was used to record and organise ideas.

The second session took place in‐person and began with a presentation of findings from the first session. Participants were then invited to reflect on the main target audiences for the forthcoming policy brief. Following the “diamond approach” [13, 26], the first session had encouraged divergent thinking (exploring ideas freely without constraints) while the second session focused on convergent thinking, narrowing down to concrete, realistic, and implementable next steps. In two separate groups, participants discussed key themes including public and political support, financing mechanisms, and practical implementation. The session concluded with a collective brainstorm on the draft script for an explainer video, which aimed to communicate the evidence and rationale for financial incentives.

Following the sessions, participants were invited to provide written feedback on draft versions of the policy brief, infographic, and explainer video. Feedback on the policy brief served as member checking to verify that it accurately reflected the Policy Lab discussions rather than as an additional source of qualitative data. The final versions (Supporting File 3) were disseminated to stakeholders in policy, insurance, public health, and relevant professional networks, and published online at www.rookvrijestart.nl/onderzoek.

#### 2.6.3 Data Collection and Analysis

Data sources included audio recordings and verbatim transcripts of the sessions, outputs generated during the pre‐mortem exercise (i.e., identified implementation risks, proposed solutions, and the prioritisation of both), and responses to the baseline questionnaire. Responses to the baseline questionnaire were analysed descriptively to characterise participants' initial views on financial incentives. A thematic analysis approach was applied to the transcripts and pre‐mortem outputs to identify implementation barriers and facilitators, as well as policy recommendations, using MAXQDA 2022 software. A deductive codebook was developed based on the Consolidated Framework for Implementation Research (CFIR) (Supporting File 4), organising insights across key domains: intervention characteristics, implementation setting, individuals, and implementation processes [27] Because the study focused on national implementation within the Dutch healthcare system, organisational implementation by providers of smoking cessation support (‘inner setting’) was closely intertwined with national financing, reimbursement, and policy structures and decisions (‘outer setting’). Therefore, the CFIR domains ‘inner setting’ and ‘outer setting’ were combined into a single domain labelled ‘implementation setting’, while retaining all underlying CFIR constructs. Additional codes were added inductively to capture relevant emergent themes. All coded segments were assigned a valence (barrier or facilitator) to assess each construct's role in the implementation process. Two researchers (SH and LvdS) independently coded the transcripts and compared results to assess intercoder agreement. Discrepancies were discussed until consensus was reached.

## 3 Results

### 3.1 Baseline Questionnaire

The results of the baseline questionnaire, presented in Supporting File 5, revealed that six of nine participants were convinced that incentives for smoking cessation are effective, two were neutral, and one was not convinced. The two neutral participants raised questions about the sustainability of effects. Opinions on using government budgets to fund the intervention were mixed, with four finding it desirable, four neutral (conditional on cost‐effectiveness and ethical considerations, according to one participant), and one opposed, questioning effectual resource allocation, unequal treatment, and precedent‐setting. Views on insurer funding were similarly divided (4 desirable, 3 neutral, 2 not desirable), with supportive respondents noting insurers' prior experience with reward programmes, while an opponent highlighted fiscal conditions and premium affordability concerns. Overall, participants acknowledged the potential of financial incentives and emphasised the importance of cost‐effectiveness and fairness.

### 3.2 Barriers and Facilitators to Implementation

The analysis identified a range of barriers and facilitators shaping the implementation of financial incentives for smoking cessation among (expectant) parents. These are structured by CFIR domains (intervention characteristics, implementation setting, individual actors, and implementation process) and detailed in Supporting File 6, including code frequencies and illustrative example quotes. The findings also informed the collaboratively developed policy brief, infographic, and explainer video (Supporting File 3).

#### 3.2.1 The Innovation

Limited awareness of the evidence base for incentive programmes was perceived as a major barrier to implementation. Some participants themselves were unfamiliar with the evidence on (cost‐)effectiveness and noted that this lack of knowledge is likely widespread among the general public. At the same time, they acknowledged convincing evidence from international trials and practice, particularly from the UK where incentives are part of standard care. Participants stressed the importance of clearly communicating this evidence, and the associated health benefits for (expectant) parents and children, to build support. Further questions were raised about the optimal incentive value, the potential need for budget caps, and the extra time and resources required for programme delivery and monitoring. Clear insight in required resources and both long and short‐term benefits was considered crucial, particularly for insurers.

I think it's really important to have the cost picture clear, and then also make the benefits visible. You have the benefits for the mother – those are long‐term – and the benefits for the child, which I think are already visible in the short term. (…) And that makes it easier to convince people.

Policymaker, Session 2

Accessible and inclusive programme design was considered equally important for successful implementation. Participants highlighted the importance of offering attractive and easy‐to‐use programme materials, rewards, and biochemical validation procedures, particularly for people with low literacy or digital skills. Robust biochemical validation was considered essential to ensure credibility and prevent gaming. Providing incentives in the form of vouchers or child‐related products rather than cash was recommended to increase feasibility and public support, and it was felt that incentives should be framed as a complementary element of evidence‐based care rather than its focus. Embedding incentives within an established, high‐quality cessation programme such as SFP was viewed as a practical way to simplify delivery, enhance trust and reach, and safeguard counselling quality. SFP was also seen as a suitable platform for piloting, allowing stakeholders to assess feasibility and demonstrate value in practice. Including participants together with their partners in the programme was also seen as a potential facilitator.

#### 3.2.2 The Implementation Setting

The broader societal and political context represents a major determinant of feasibility for incentive‐based cessation programmes. Stakeholders thought that a lack of public and political support would be the central barrier. Critiques included the view that smoking was seen as a matter of individual responsibility and that incentives are patronising, unfair toward non‐smokers, or ethically questionable when targeting only specific groups like (expectant) parents. Media attention to gaming or negative public debate might further undermine political and societal support.

In the Netherlands, when it comes to money, we tend to be very suspicious about these kinds of things.

Participating researcher, Session 1

At the same time, participants suggested that support could be strengthened through communication campaigns emphasising that smoking is an addiction and therefore a medical condition that warrants treatment, and by making explicit that the high societal costs of smoking are borne collectively. Public acceptability was also seen as dependent on the form of incentives (e.g., vouchers or child‐related products) and the fairness of the system, with safeguards against gaming. Finally, lobbying and collective pressure from insurers' umbrella organisations and advocacy groups such as the Netherlands Lung Foundation (*Longfonds*), aligned with the National Prevention Agreement [28] were seen as potential drivers of policy commitment and coordinated action.

Legal and financial structures were viewed as additional major constraints to implementation. According to participating policymakers, financial incentives do not fall under the legal definition of healthcare within the Dutch Health Insurance Act. The law also prohibits unequal treatment, raising concerns about selective eligibility for incentives. Inclusion in the basic insurance package was therefore regarded as unlikely in the short term. Participants discussed alternative strategies such as financing through insurers' prevention budgets or supplementary insurance schemes. The example of ‘*Afvallen met Afspraken*’ (*Weight Loss with Commitments*), an insurer‐funded incentive pilot programme outside the basic package, was highlighted as a potential precedent [29] Still, insurer buy‐in was seen as uncertain due to (1) fiscal challenges; (2) the delayed return on investment typical of prevention, which may even benefit another insurer when individuals switch insurance plans; and (3) the diffuse nature of prevention benefits within insurers' own organisations, which complicates internal budgeting. Other potential funding sources mentioned included subsidies from municipalities, national prevention funds, the national government, or municipal insurance schemes for low‐income groups. Concerns about structural embedding remained, particularly if funding relied on temporary subsidies.

#### 3.2.3 Individuals and Their Roles in Implementation

Barriers and facilitators to implementation varied between stakeholder groups (i.e., healthcare professionals, providers of smoking cessation support, (expectant) parents, policymakers, and insurers), each with different roles in the cessation support system. For healthcare professionals and providers of smoking cessation support, awareness and adequate resources were seen as key conditions for successful delivery. Integration of incentives into existing structures, such as the SFP programme, could increase awareness and reach, and provide training and programme materials to support professionals in delivering high‐quality, accessible care. For recipients, (expectant) parents, financial incentives were perceived as aligning with their practical needs, particularly when vouchers could be used flexibly for goods or services of their choice. Barriers included low health and digital literacy, as well as stigma that may prevent individuals from disclosing smoking or seeking support. Participants recommended an accessible programme design, validation procedures at familiar healthcare locations, and clear communication in multiple languages to ensure equitable participation.

Risks include logistical challenges with distributing the rewards and CO monitors, or when parents are unable to complete the validation.

Provider of smoking cessation support, Session 1

Politicians' motivation was seen as depending on prevailing political priorities and public opinion, whereas policymakers were generally more supportive of prevention‐oriented approaches but acted within a policy mandate shaped by existing legislative and budgetary frameworks and the political climate. Politicians could be reminded of their responsibility to address smoking as a public health issue and convinced through clear evidence of cost‐effectiveness, while also being prepared to address public debate. Insurers' motivation to fund incentives similarly depended on clear evidence and confidence in cost‐effectiveness. Since there is no legal obligation for prevention, investment in incentive‐based programmes would require voluntary commitment. However, participants noted that insurers might also view participation as a strategic opportunity to attract new clients and strengthen their social profile, while collective action across insurers could enable fair and sustainable sharing of risks and benefits.

#### 3.2.4 The Implementation Process

Participants stressed the importance of bringing together all relevant actors to clarify roles, responsibilities, and opportunities. Learning from existing initiatives was also recommended. Tailoring strategies to the needs of (expectant) parents in vulnerable circumstances and engaging a wide range of relevant stakeholders, including general practitioners, social workers, and municipalities, were considered critical for successful implementation through reaching those most in need. Piloting was seen as a key facilitator to convince funders and policymakers, with temporary funding from prevention funds or insurers suggested as a starting point. Campaigns were identified as central: messaging should be tailored to various stakeholder groups, including politicians, insurers, healthcare professionals, (expectant) parents, and the broader public. Communication should be clear and convincing, and some participants suggested avoiding the term “reward” due to negative connotations, proposing alternatives such as “voucher” or “gesture of support”.

If you focus more on community organisations – neighbourhood or child centres, for example – and make sure they have the right information, they can reach the target group directly. They often already have someone who speaks the language and can help parents sign up, and so on. I think that's mainly what's important.

Parent, Session 1

## 4 Discussion

This study provides new insights into the complex interplay of social, political, and structural factors shaping the implementation of financial incentives for smoking cessation among (expectant) parents in the Netherlands. While incentives are well‐established as an effective and cost‐effective intervention [5] this study highlights that their successful implementation depends on building public and political legitimacy, ensuring sustainable and legally feasible financing mechanisms, and designing programmes that are practical, inclusive, and trusted.

### 4.1 Public and Political Legitimacy

A central finding was that stakeholders perceived limited public and political support as a major barrier to implementation of financial incentives. Consistent with earlier studies, participants highlighted limited awareness of incentive effectiveness [30, 31] and ethical concerns about fairness and “rewarding” unhealthy behaviour [7, 31, 32, 33] as key determinants of limited acceptability. Such concerns can be mitigated through ethical reflection and early public engagement during intervention design [9, 34], by framing incentives as part of evidence‐based treatment for addiction rather than as rewards for individual choice [34, 35], and through tailored communication that highlights both the health and societal benefits, particularly the intergenerational protection of children [34, 36]. Tailored public campaigns are supported by evidence showing that information‐based interventions can substantially increase acceptability [30] and are reflected in the communication strategy of the NHS England smoke‐free pregnancy incentive programme [37].

### 4.2 Financing

Financing and associated legal frameworks emerged as critical challenges. Under the Dutch Health Insurance Act, financial incentives fall outside the legal definition of healthcare, which currently precludes reimbursement through the basic insurance package. This finding illustrates that successful implementation depends not only on evidence of effectiveness but also on financing arrangements and regulatory frameworks that enable preventive interventions to be funded and implemented, as also noted in cross‐national comparisons [5, 9]. While England benefits from centralised commissioning within the National Health Service, countries with insurance‐based systems such as the Netherlands face fragmented financing, diffuse accountability, and strict care definitions. Similar ambiguities exist elsewhere, for instance, U.S. Medicaid programmes restrict patient incentives unless specific waivers apply [38] Beyond these legal considerations, our findings highlight broader financing challenges common to preventive interventions, with delayed returns on investment and the fragmented distribution of benefits across organisations and sectors. These findings align with recent work arguing that many healthcare systems lack sustainable financing structures for preventive interventions and may require reimbursement frameworks that better accommodate evidence‐based prevention [39]

Participants also proposed pragmatic pathways forward, such as piloting incentive programmes through prevention budgets or supplementary insurance to demonstrate real‐world feasibility and cost‐effectiveness. Yet whether additional pilots are warranted remains debatable, given the extensive international evidence on both effectiveness [5] and implementation [9, 40], as well as prior (Dutch) pilot experiences [33, 41, 42]. While stakeholders viewed pilots as a pragmatic means to build trust among funders and policymakers, the key challenge lies in translating temporary, project‐based initiatives into structurally financed programmes. Addressing this requires political commitment alongside collaboration between policymakers and other stakeholders to develop legally and financially viable models for prevention‐oriented care.

### 4.3 Programme Design

While sustainable financing forms the structural foundation for implementation, programme design determines practical feasibility and equity. Embedding incentives within existing programmes such as SFP was viewed as a good strategy to enhance feasibility, trust, and reach, consistent with evidence identifying integration into existing care models as a facilitator of implementing financial incentive programmes [9] Participants emphasised accessible and user‐centred design to ensure equity, particularly for (expectant) parents with low literacy or digital skills [9, 11, 43, 44]. For example, participants highlighted the importance of tailoring referral strategies to (expectant) parents' needs and local contexts, including through trusted community organisations. These findings are consistent with previous co‐creation work and reinforce the value of continued participatory approaches [45] Selective targeting, such as limiting eligibility to pregnant women, may affect legal feasibility and perceived fairness [7, 46]. To improve fairness and acceptability, participants suggested premium discounts or child‐related incentives, although studies show that delayed incentives like premium discounts are generally less effective than immediate ones [47, 48]. Finally, several participants stressed the importance of engaging social networks, for instance by involving partners to enhance motivation and retention, reflecting both scientific evidence and current UK practice [9, 37].

### 4.4 Implications

Implementing financial incentives requires coordinated action across policy, financing, and delivery levels. Policymakers and insurers should jointly explore sustainable funding models, such as through prevention budgets or collective insurer agreements, and clear legal pathways for prevention‐oriented care. Public communication should emphasise fairness, collective benefit, and child health protection, while implementation efforts should prioritise low‐barrier, inclusive programme design within trusted cessation services. Further economic modelling and participatory preference studies are needed to determine context‐appropriate incentive values and financing arrangements [5, 43], as well as on how informational campaigns influence public and political support.

### 4.5 Strengths and Limitations

To our knowledge, this is the first empirical study to co‐develop implementation recommendations for financial incentives for smoking cessation with policymakers, insurers, healthcare professionals, researchers, and end‐users, using a policy lab approach grounded in implementation theory. The inclusion of diverse stakeholders and translation to actionable recommendations in accessible formats (policy brief, infographic and video) enhanced the relevance and practicality of the findings. The pre‐mortem exercise proved valuable for fostering critical reflection and surfacing hidden barriers. Future labs may benefit from hybrid formats, as attendance in the second session was lower due to logistical constraints. Although the second session focused on prioritising recommendations generated during the first session, reduced attendance may have limited the diversity of perspectives during this convergent phase. Individual follow‐up discussions and participant review of the draft policy brief helped mitigate this risk. As in all qualitative policy research, insights are context‐specific but reveal mechanisms, including legitimacy, framing, and cross‐sectoral exploration of financing options, that are relevant across settings and populations.

## 5 Conclusion

This study illustrates that effective implementation of financial incentives for smoking cessation extends far beyond evidence of clinical efficacy. Success depends on establishing legitimacy, aligning financing mechanisms with existing health system structures, and designing interventions that are equitable and trusted. These findings provide practical guidance for policymakers seeking to embed incentive‐based approaches within cessation care and broader public health policy.

## Author Contributions

**Linda van der Spek:** conceptualisation, funding acquisition, investigation, formal analysis, writing – original draft, writing – review and editing. **Niek Mouter:** funding acquisition, investigation, supervision, writing – review and editing. **Tessa Scheffers‐van Schayck:** conceptualisation, funding acquisition, investigation, writing – review and editing. **Leonieke J. Breunis:** funding acquisition, writing – review and editing. **Hans van Kippersluis:** conceptualisation, funding acquisition, investigation, supervision, writing – review and editing. **Stijn Hooijman:** formal analysis, writing – review and editing. **Jasper V. Been:** conceptualisation, funding acquisition, investigation, supervision, writing – review and editing.

## Ethics Statement

Ethical approval for this study was obtained from the Erasmus MC Non‐WMO Review Committee, which confirmed that the study was not subject to the Dutch Medical Research Involving Human Subjects Act (WMO) and approved the study procedures (MEC‐2024‐0309). All participants received written information about the study and provided written informed consent prior to participation. Participants received reimbursement of travel expenses and a compensation of €50 per session for their time and contribution.

## Conflicts of Interest

The authors declare no conflicts of interest.

## Data Protection

All procedures complied with the General Data Protection Regulation (GDPR) and the Dutch GDPR Implementation Act (UAVG). Audio recordings and transcripts were securely stored and anonymised prior to analysis.

## AI Use

Throughout the writing process, ChatGPT (OpenAI, GPT‐5.2) was used for language editing and improving clarity. All AI‐assisted text was reviewed and revised by the authors to ensure accuracy.

## Supporting information


Supporting File 1

Supporting File 2

Supporting File 3

Supporting File 4

Supporting File 5

Supporting File 6

## Data Availability

Anonymised data are available from the corresponding author upon reasonable request.
