# Supplementary material for: Stakeholder Perspectives on National Implementation of Financial Incentives for Smoking Cessation Before, During and After Pregnancy: A Policy Lab Study
Source: Health Expect. 2026 Oct 12;29(5):e70893. doi: 10.1111/hex.70893 (PMC13656341; doi:10.1111/hex.70893)
Supplement: Supplementary file 2 [file HEX-29-e70893-s003.pdf]

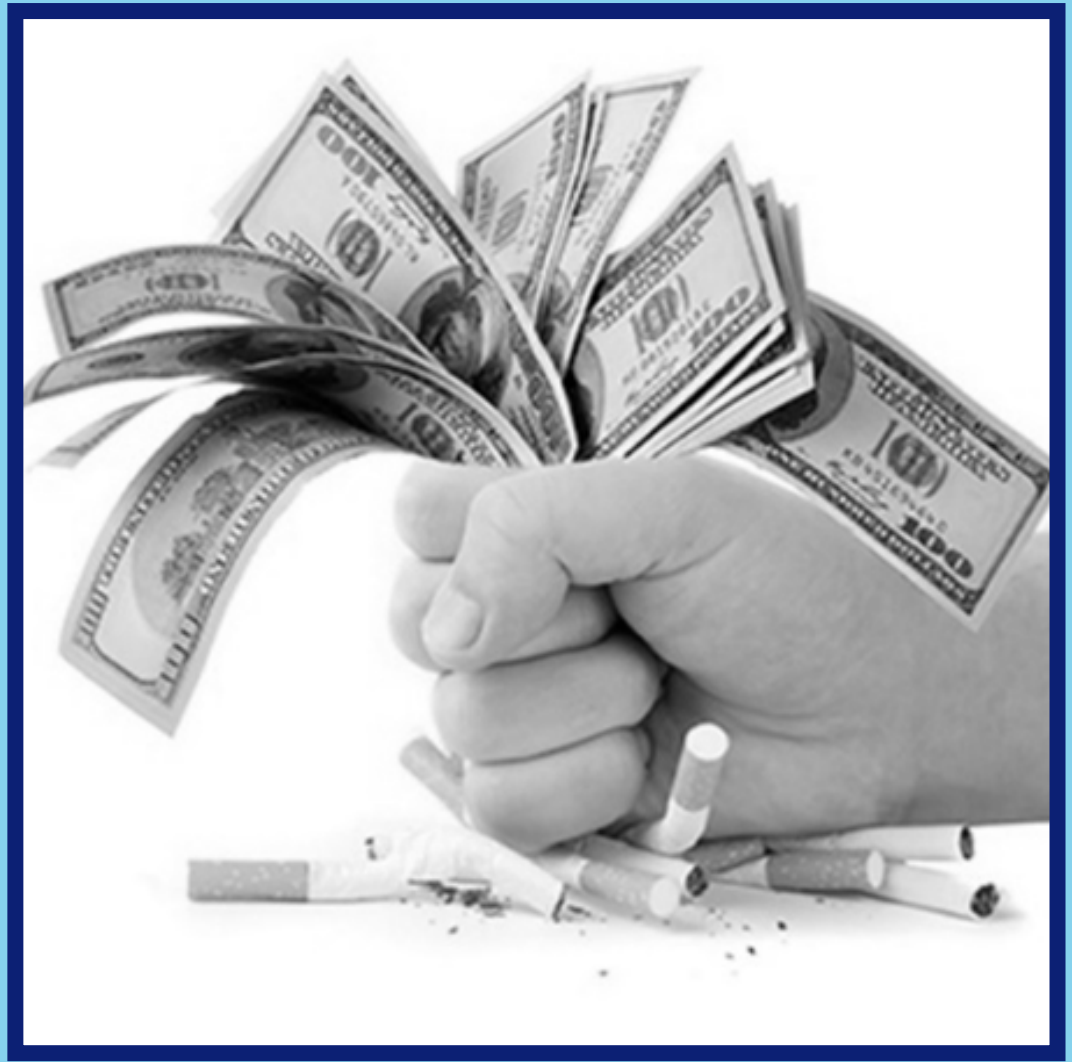

Financial incentives for smoking cessation among (expectant) parents

By Linda van der Spek (lspek@trimbos.nl), Hans van Kippersluis, Leonieke Breunis, Niek Mouter, Tessa Scheffers-van Schayck, and Jasper Been.

BACKGROUND

How many (expectant) parents smoke?

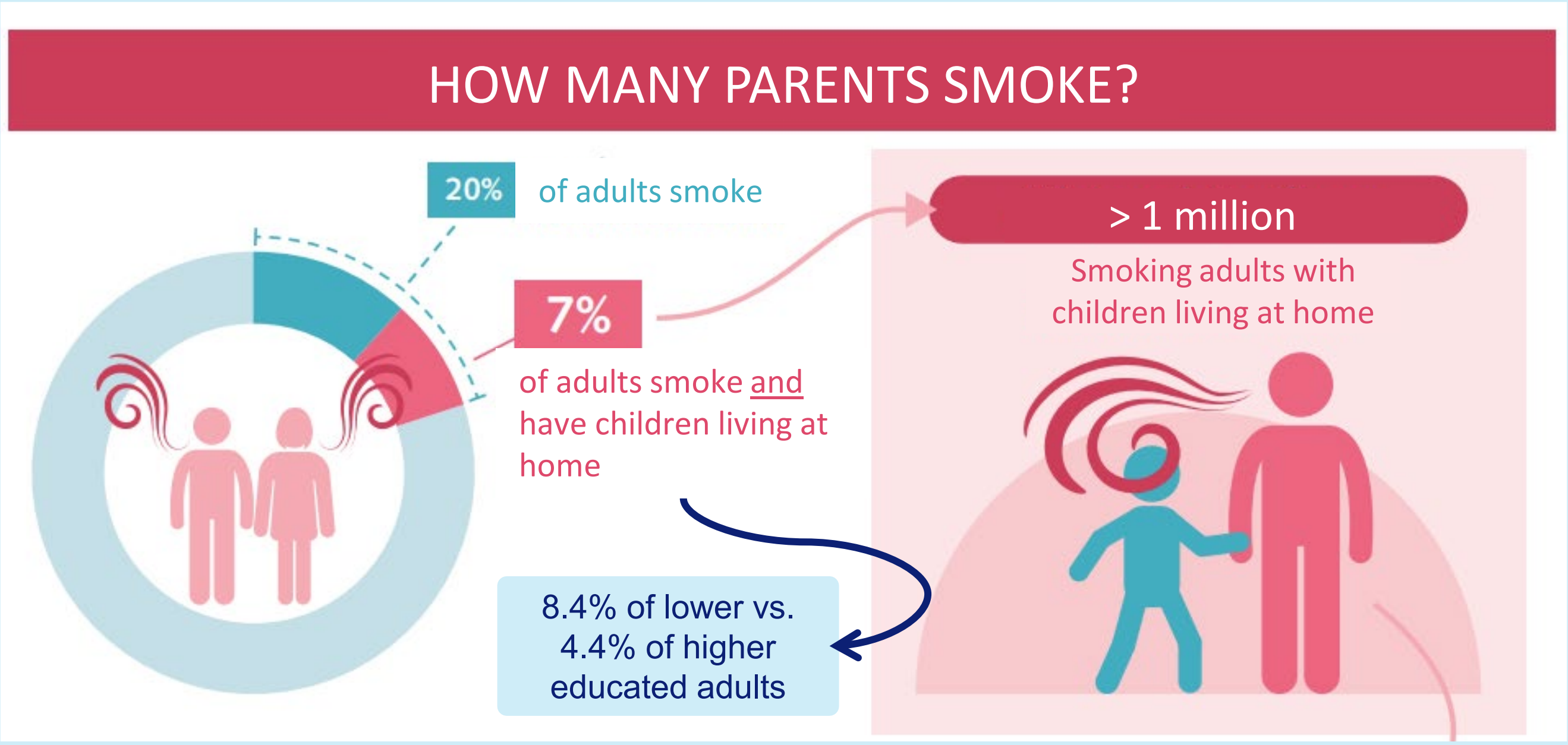

National Health Survey/Lifestyle Monitor by CBS, RIVM and the Trimbos Institute, 2020.

&

Smoking ↔ socioeconomic inequalities
(O'Connell et al., 2022; Gregoraci et al., 2017)

20.3% of **lower** educated
vs. 2.5% of **higher** educated
Dutch women smoked at any moment during their pregnancy
(Scheffers-van Schayck et al, 2024)

Erasmus MC
Sophia Kinderziekenhuis

BACKGROUND
The Smoke-Free Parents program

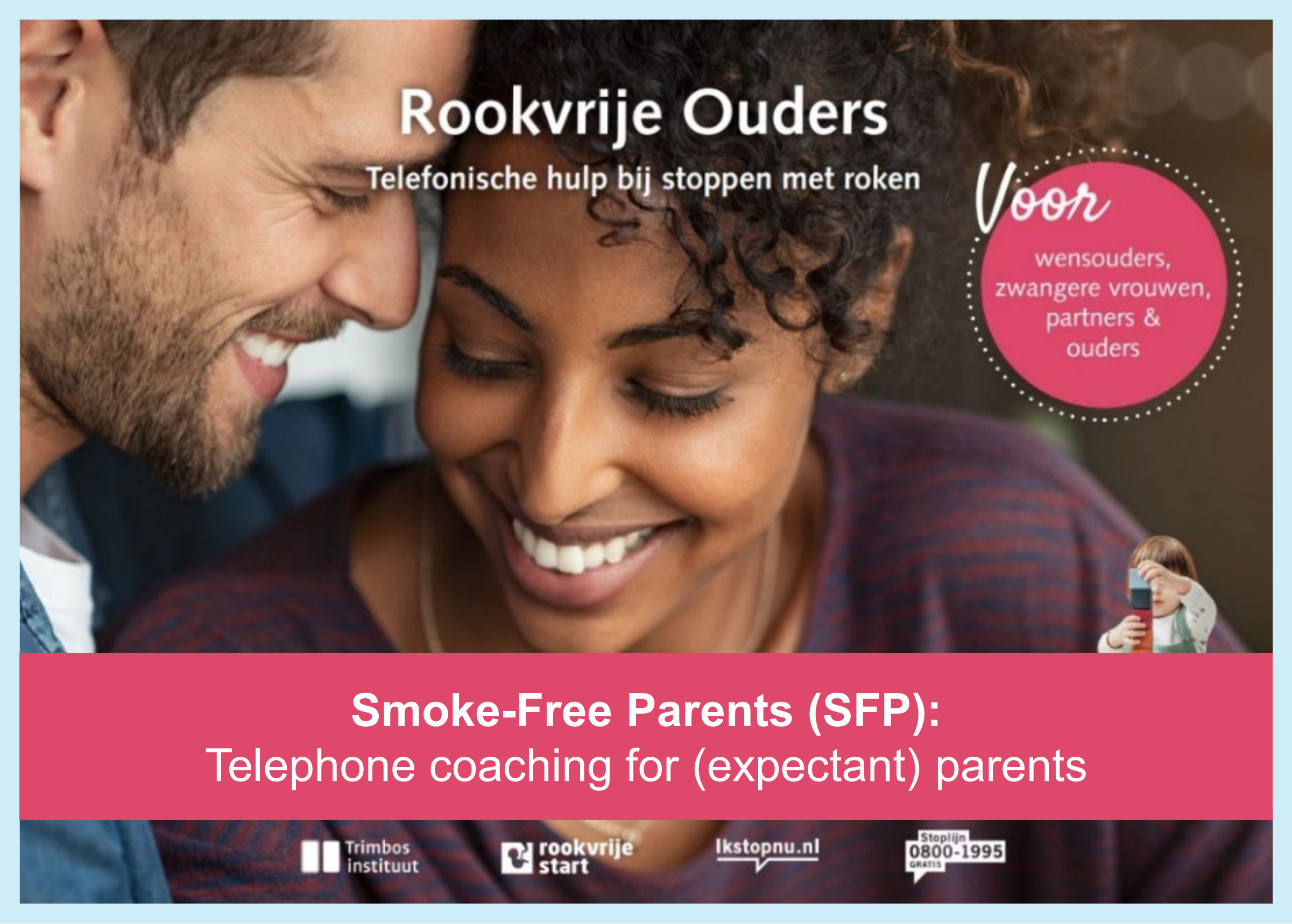

Reimbursed from the basic insurance, exempt from deductible.

Available throughout the Netherlands since 2019

Motivational interviewing + interactive magazine

> 600 registrations in 2023

> 50% actually starts the coaching trajectory

Effectieve
(Schuck et al., 2014; Scheffers-van Schayck et al., 2019)

Erasmus MC Sophia Kinderziekenhuis

BACKGROUND

What are financial incentives?

'Financiële beloningen' often referred to in the scientific literature as 'Financial incentives' or 'Contingency Management'. They are rewards (such as money, vouchers or products) for behavioural change.

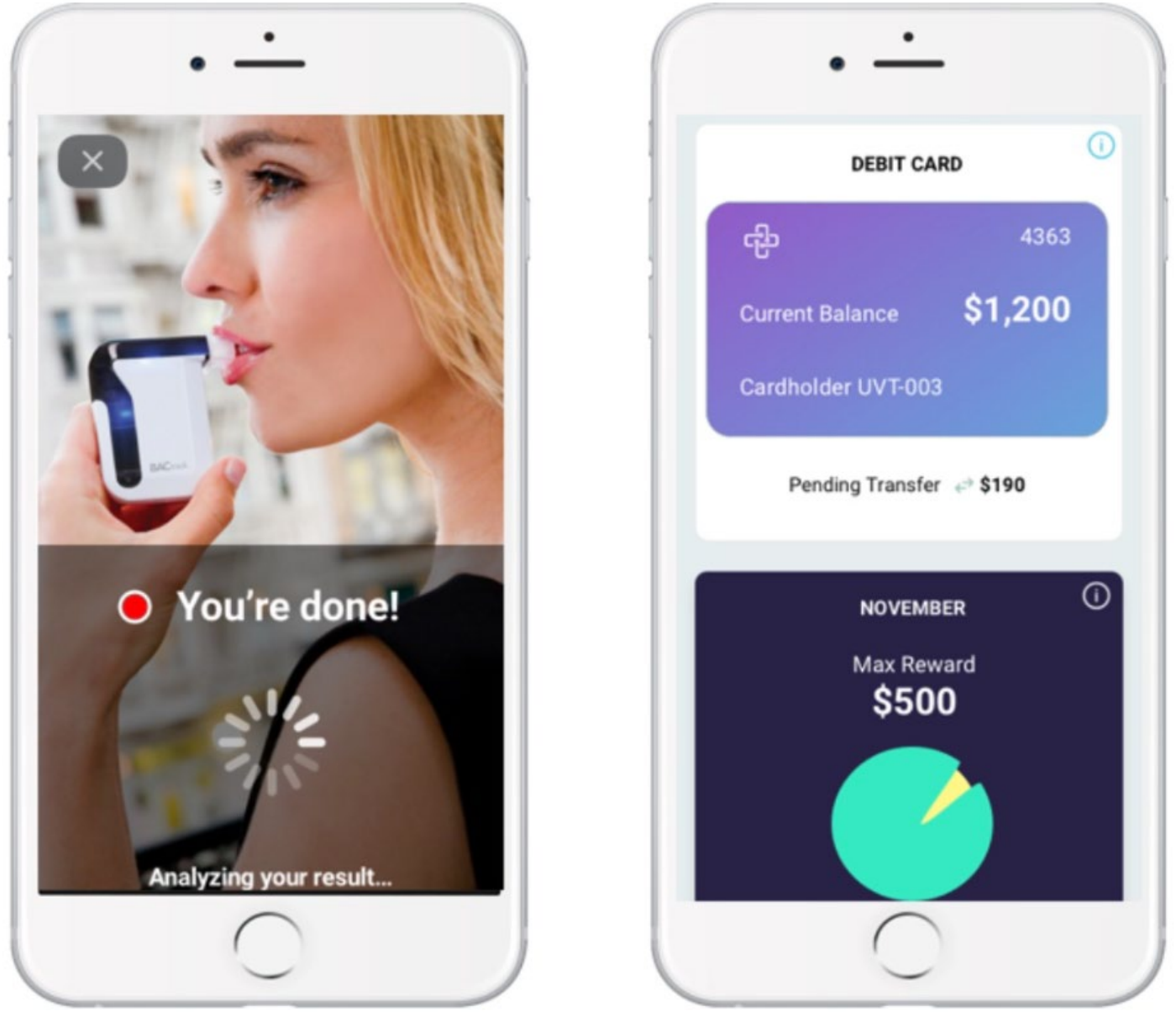

Biochemical validation shows whether a participant is smoke-free and deserves a reward, for example by means of a CO breath test. Source: Nicole Twohig, The University of Vermont.

Rewards come in many forms, as outlined in the framework of Adams et al. (2014):

Domain	Dimension	Example
Direction	Positive reward	Welfare benefits increased for confirming all immunisations are up to date in children (Lawrence et al., 2004)
	Avoidance of penalty	Welfare benefits decreased for failing to confirm all immunisations are up to date in children (Minkovitz et al., 1999)
Form	Cash	Cash reward for weight loss (Jeffery et al., 1983)
	Vouchers for range of goods/services	Grocery voucher for smoke-free breath test (Ballard & Radley, 2009)
	Specific goods/service	MP3 player for weight loss (Petry et al., 2011)
Magnitude	Continuous (not categorical) variable	Total value of incentive available to participants; ideally considered in relation to individual socio-economic circumstances
Certainty	Certain	Grocery voucher for smoke-free breath test (Ballard & Radley, 2009)
	Certain chance	Chance to draw from bowl of 500 tickets, 250 of which are associated with prizes, for weight loss (Petry et al., 2011)
	Uncertain chance	Entered into lottery to win holiday for smoke-free breath test (Cahill & Perera, 2011)
Target	Process	Shopping vouchers for attendance at routine outpatient drug rehabilitation sessions (Carroll et al., 2006)
	Intermediate	Cash reward for attending supervised walks to achieve weight-loss (Jeffery et al., 1998)
	Outcome	Shopping vouchers for drug-free urine (Carroll et al., 2006)
	Proxy measures of behaviour	Cash reward proportionate to weight loss (Jeffery et al., 1983)
Frequency	All instances incentivised	Cash reward based on number of physically active minutes per week (Finkelstein et al., 2008)
	Some instances incentivised	Grocery voucher for smoke-free breath test, measured once per week (Ballard & Radley, 2009)
Immediacy	Continuous (not categorical) variable	How soon reward is received after behaviour is performed
Schedule	Fixed	Weight loss reward at a fixed rate per pound lost (or maintained as lost) per month (Relton et al., 2011)
	Variable	Drug users received $25 shopping vouchers for the first treatment session attended, increasing by $5 for each additional consecutive session attended (Carroll et al., 2006)
Recipient	Individual	Cash reward proportionate to weight loss achieved by individual participant (Jeffery et al., 1983)
	Group	Cash proportionate to average weight loss in group of participants (Jeffery et al., 1983)
	Significant other	Cash to nominated significant other for weight loss in participant (Jeffery et al., 1983)

Erasmus MC
Sophia Kinderziekenhuis

BACKGROUND

Financial incentives are effective

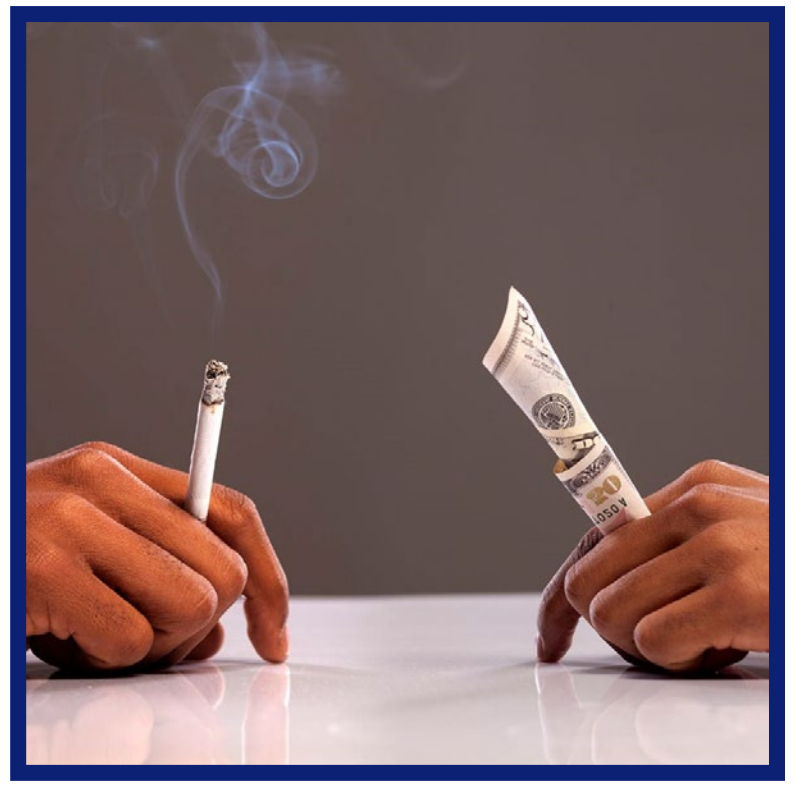

A systematic review and meta-analysis

door LS Kock · 2023 — **Pregnant women receiving incentives** are **twice** as **likely** to **achieve smoking abstinence during pregnancy suggesting** this **intervention should** be ...

Effectiveness of smoking cessation interventions among ...

door A Vila-Farinas · 2023 · Geciteerd door 4 — According to the results of the current meta-analysis, incentives appear to be the most effective intervention for smoking cessation (high...

Psychosocial interventions for supporting women to stop ...

door C Chamberlain · 2017 · Geciteerd door 760 — **High-quality evidence suggests incentive-based interventions** are **effective** when **compared** ... (**four studies**; **RR 2.36**, **95**...

Incentives for smoking cessation

door C Notley · 2025 — Overall, our conclusion from this latest review update remains that there is **high-certainty** evidence that **incentives** improve **smoking cessation**

Two to three times as effective as standard care or psychosocial help

The **effects last**, even after rewards and after pregnancy (Kock et al., 2023; Notley et al., 2025)

With rewards, **37%** of women were smoke-free at the end of pregnancy, compared to 16% in interventions without incentives (Chamberlain et al., 2017).

BACKGROUND

Financial incentives are cost-effective

Cost-effectiveness was demonstrated in the United Kingdom and the United States (NICE, 2021; McMeekin et al., 2023; Shepard et al., 2022).

Financial incentives for quitting smoking in pregnancy

door N McMeekin · 2023 · Geciteerd door 4 — **Offering up to £400 financial incentives**, in addition to usual care, to support pregnant women to stop smoking appears to be highly cost-...

- **Highly cost-effective:**
- Cost savings (for mother and child throughout their lives) and an improvement in quality of life (QALYs).
- This intervention saves costs instead of having to pay extra for it. In the United Kingdom, the willingness to pay is £20,000 per QALY won; in the Netherlands, this is between €20,000 and €80,000.

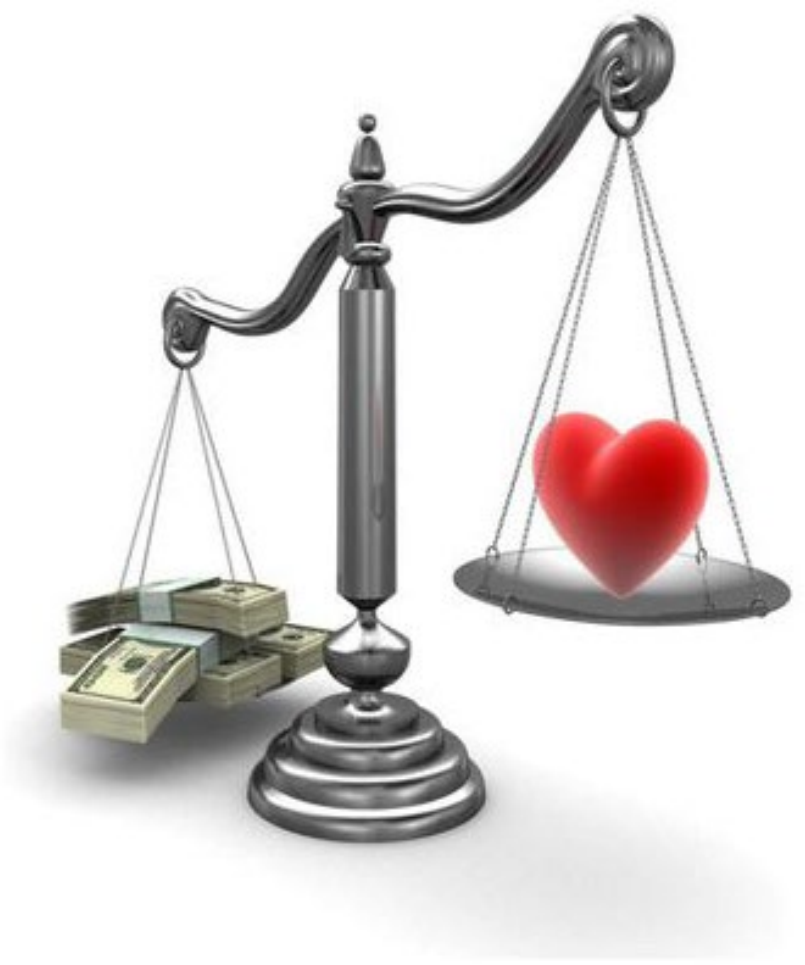

Erasmus MC Sophia Kinderziekenhuis

BACKGROUND

Standard practice in the United Kingdom

The UK government has announced that by the end of 2024, all pregnant women who smoke will be offered financial rewards (link).

NICE guideline 2021:

These recommendations are for providers of stop-smoking support.

1.20.12 In addition to NRT and behavioural support, offer voucher incentives to support women to stop smoking during pregnancy, as follows:

RESEARCH IN THE NETHERLANDS

How have financial incentives been implemented and researched in the Dutch context?

Pilot study by Breunis et al., 2023:

- Group sessions, online platform and rewards (up to €185);
- 39 smoking women (preconception/pregnant);
- 28% quit smoking, 33% dropped out;
- Appreciation for personal approach and biochemical validation;
- Appropriate intervention, but recruitment and dropout require attention.

Cluster RCT by Van den Brand et al., 2018:

- Incentives (up to €350) with group coaching;
- In 61 companies with 604 employees and their partners;
- After 12 months, abstinence in the intervention group was significantly higher (41%) than in the control group (26%);
- Support employers in recruitment; convince them that non-smoking employees do not consider the incentives to be unfair and that it is cost-effective (Van den Brand et al., 2019).

Pilot study by Kroder et al., 2022:

- Healthcare professionals (n=5) and pregnant women (n=9) found the incentives (up to €575) helpful;
- Implementation was feasible;
- Professionals were enthusiastic, despite initial concerns about fairness towards non-smoking pregnant women;
- Most participants had a lower SES.

Planned in 2025: Pilot study Smoke-Free Parents:

- Expansion of the national telephone coaching Smoke-Free Parents with financial incentives (up to €400), self-monitoring, social support and aftercare;
- Max. 200 participants;
- Quantitative and qualitative evaluation of reach, effectiveness and implementation.

ETHICAL FRAMEWORK

Criteria for identifying and addressing unethical consequences

Beneficence
1: Is the intervention **effective**?
2: Does the reward support a **healthy** lifestyle?
3: Is the intervention **motivating** according to the target group?

Respect for autonomy
6: Is **personal autonomy** respected?
7: In addition to extrinsic motivation, is **intrinsic motivation** also increased?
8: Is **privacy** respected?

Nonmaleficence
4: Does the intervention avoid stimulating unhealthy behaviors?
5: Is the risk of negative attitudes towards the participants and the intervention minimized?

Justice
9: Is the intervention **fair** to non-smokers?
10: Are the rewards given to those **who deserve them**?
11: Is the intervention **cost-effective**?
12: Does the intervention improve the health of the **most vulnerable**?

(Breunis et al., 2020)

"None of the included pregnancy trials reported on harms or unintended consequences of the interventions."
(Cochrane review Notley et al., 2019)

Erasmus MC
Sophia Kinderziekenhuis

SOCIAL SUPPORT

Statement: "Giving incentives (such as vouchers) to pregnant women who quit smoking is an appropriate additional tool."

	Agree	Neutral	Disagree
**France, 2018**	55%	3%	42%
**United Kingdom, 2014**	41%	17%	42%
**Netherlands, 2023**	29%	35%	36%

Bronnen: Berlin et al., 2018; Hoddinott et al., 2014; Dutch Public support survey on tobacco control policy, 2024.

Erasmus MC
Sophia Kinderziekenhuis

OUR SYSTEMATIC REVIEWS

1. What hinders and promotes the implementation of financial incentives as part of smoking cessation interventions?

- Impeding factors: lack of alignment with the context and resources of participants, challenges in recruitment and retention, limited reliability of biochemical validation, and high time and resource burden.
- Promoting factors: ensuring suitability, accessibility and usability.

2. What are the effective elements of smoking cessation interventions for (expectant) parents with a lower SES?

- The comparison between the applied behavioural change techniques in effective and ineffective interventions showed that monitoring & feedback and rewarding were the techniques that made the difference.
- Increasing knowledge and providing social support were the most common in general.

The scientific publications are currently being prepared.

Erasmus MC
Sophia Kinderziekenhuis

REFERENCES

- Adams, J., Giles, E. L., McColl, E., & Sniehotta, F. F. (2014). Carrots, sticks and health behaviours: a framework for documenting the complexity of financial incentive interventions to change health behaviours. *Health Psychology Review*, *8*(3), 286–295. https://doi.org/10.1080/17437199.2013.848410
- Berlin, N., Goldzahl, L., Bauld, L., Hoddinott, P., & Berlin, I. (2018). Public acceptability of financial incentives to reward pregnant smokers who quit smoking: a United Kingdom–France comparison. *European Journal of Health Economics*, *19*(5), 697–708. https://doi.org/10.1007/s10198-017-0914-6
- Breunis, L. J., Been, J. V., De Jong-Potjer, L., Steegers, E. A. P., De Beaufort, I. D., De Kroon, M. L. A., & Ismaili M'Hamdi, H. (2020). Incentives for smoking cessation during pregnancy: An ethical framework. *Nicotine and Tobacco Research*, *22*(9), 1553–1559. https://doi.org/10.1093/ntr/ntz231
- Breunis, L. J., de Kroon, M. L. A., de Jong-Potjer, L. C., Steegers, E. A. P., & Been, J. V. (2023). Piloting a complex intervention to promote a tobacco and alcohol-free pregnancy: the Smoke and Alcohol Free with EHealth and Rewards (SAFER) pregnancy study. *BMC Pregnancy and Childbirth*, *23*(1), 1–14. https://doi.org/10.1186/s12884-022-05320-8
- Chamberlain, C., O'Mara-Eves, A., Porter, J., Coleman, T., Perlen, S. M., Thomas, J., & Mckenzie, J. E. (2017). Psychosocial interventions for supporting women to stop smoking in pregnancy. *Cochrane Database of Systematic Reviews*, *2017*(2). https://doi.org/10.1002/14651858.CD001055.pub5
- Gregoraci, G., van Lenthe, F. J., Artnik, B., Bopp, M., Deboosere, P., Kovács, K., Looman, C. W. N., Martikainen, P., Menvielle, G., Peters, F., Wojtyniak, B., de Gelder, R., & Mackenbach, J. P. (2017). Contribution of smoking to socioeconomic inequalities in mortality: A study of 14 European countries, 1990–2004. *Tobacco Control*, *26*(3), 260–268. https://doi.org/10.1136/tobaccocontrol-2015-052766
- Hoddinott, P., Morgan, H., MacLennan, G., Sewel, K., Thomson, G., Bauld, L., Yi, D., Ludbrook, A., & Campbell, M. K. (2014). Public acceptability of financial incentives for smoking cessation in pregnancy and breast feeding: A survey of the British public. *BMJ Open*, *4*(7). https://doi.org/10.1136/bmjopen-2014-005524
- Kock, L. S., Erath, T. G., Coleman, S. R. M., Higgins, S. T., & Heil, S. H. (2023). Contingency management interventions for abstinence from cigarette smoking in pregnancy and postpartum: A systematic review and meta-analysis. *Preventive Medicine*, *July*, 107654. https://doi.org/10.1016/j.ypmed.2023.107654
- Kroder, T. A., Peters, L. L., Roggeveld, A. L., Holtrop, M., Harshagen, L., Klein, L. M., & Erwich, J. J. H. M. (2022). Smoking cessation in pregnant women using financial incentives: a feasibility study. *BMC Pregnancy and Childbirth*, *22*(1), 1–14. https://doi.org/10.1186/s12884-022-05292-9
- McMeekin, N., Sinclair, L., Robinson-Smith, L., Mitchell, A., Bauld, L., Tappin, D. M., & Boyd, K. A. (2023). Financial incentives for quitting smoking in pregnancy: Are they cost-effective? *Addiction*, *February*, 1–12. https://doi.org/10.1111/add.16176
- National Institute for Health and Care Excellence. (2021). *Tobacco: Preventing Uptake, Promoting Quitting and Treating Dependence. [I] Evidence reviews for incentives during pregnancy*. National Institute for Health and Care Excellence (NICE).
- National Institute for Health and Care Excellence. (2023). *Tobacco: preventing uptake, promoting quitting and treating dependence: Rationale and impact*. https://www.nice.org.uk/guidance/ng209
- Notley, C., Gentry, S., Livingstone-Banks, J., Bauld, L., Perera, R., & Hartmann-Boyce, J. (2019). Incentives for smoking cessation. *Cochrane Database of Systematic Reviews*, *2019*(7). https://doi.org/10.1002/14651858.CD004307.pub6
- O'Connell, N., Burke, E., Dobbie, F., Dougall, N., Mockler, D., Darker, C., Vance, J., Bernstein, S., Gilbert, H., Bauld, L., & Hayes, C. B. (2022). The effectiveness of smoking cessation interventions for socio-economically disadvantaged women: a systematic review and meta-analysis. *Systematic Reviews*, *11*(1), 1–22. https://doi.org/10.1186/s13643-022-01922-7
- Scheffers-van Schayck, T., Ramaker, V., Van Engen, M., Morren, K., & Tuithof, M. (2024). *Monitor Middelengebruik en Zwangerschap 2023. Middelengebruik van vrouwen en hun partners voor, tijdens en na de zwangerschap.* https://www.trimbos.nl/aanbod/webwinkel/af2186-monitor-middelengebruik-en-zwangerschap-2023/
- Shepard, D. S., Slade, E. P., Nighbor, T. D., DeSarno, M. J., Roemhildt, M. L., Williams, R. K., & Higgins, S. T. (2022). Economic analysis of financial incentives for smoking cessation during pregnancy and postpartum. *Preventive Medicine*, *165*(PB), 107079. https://doi.org/10.1016/j.ypmed.2022.107079
- van den Brand, F. A., Magnée, T., de Haan-Bouma, L., Barendregt, C., Chavannes, N. H., van Schayck, O. C. P., & Nagelhout, G. E. (2019). Implementation of financial incentives for successful smoking cessation in real-life company settings: A qualitative needs assessment among employers. *International Journal of Environmental Research and Public Health*, *16*(24). https://doi.org/10.3390/ijerph16245135
- van den Brand, F. A., Nagelhout, G. E., Winkens, B., Chavannes, N. H., & van Schayck, O. C. P. (2018). Effect of a workplace-based group training programme combined with financial incentives on smoking cessation: a cluster-randomised controlled trial. *The Lancet Public Health*, *3*(11), e536–e544. https://doi.org/10.1016/S2468-2667(18)30185-3
- Van Westen-Lagerweij, N., Van den Eeden, N., & Bommelé, J. (2022). *Meeroken door kinderen. Feiten en cijfers 2020.* Trimbos-instituut. https://www.trimbos.nl/wp-content/uploads/2022/05/INF119-Meeroken-door-kinderen.pdf
- Vila-Farinas, A., Perez-Rios, M., Montes-Martinez, A., Ruano-Ravina, A., Forray, A., Rey-Brandariz, J., Candal-Pedreira, C., Fernández, E., Casal-Acción, B., & Varela-Lema, L. (2024). Effectiveness of smoking cessation interventions among pregnant women : An updated systematic review and meta-analysis. *Addictive Behaviors*, *148*(August 2023). https://doi.org/10.1016/j.addbeh.2023.107854
