# Supplementary material for: Stakeholder Perspectives on National Implementation of Financial Incentives for Smoking Cessation Before, During and After Pregnancy: A Policy Lab Study
Source: Health Expect. 2026 Oct 12;29(5):e70893. doi: 10.1111/hex.70893 (PMC13656341; doi:10.1111/hex.70893)
Supplement: Supplementary file 3 [file HEX-29-e70893-s006.pdf]

RESEARCH ON SMOKING CESSATION SUPPORT

Vouchers to support (expectant) parents to quit smoking

28 Every day in the Netherlands, 28 babies enter the world already affected by harmful tobacco smoke in the womb.

These children neither chose nor can prevent this. They deserve a healthy start – free from preterm birth, asthma, and other serious health problems.

What if we could offer even better support to (expectant) parents who want to quit smoking?

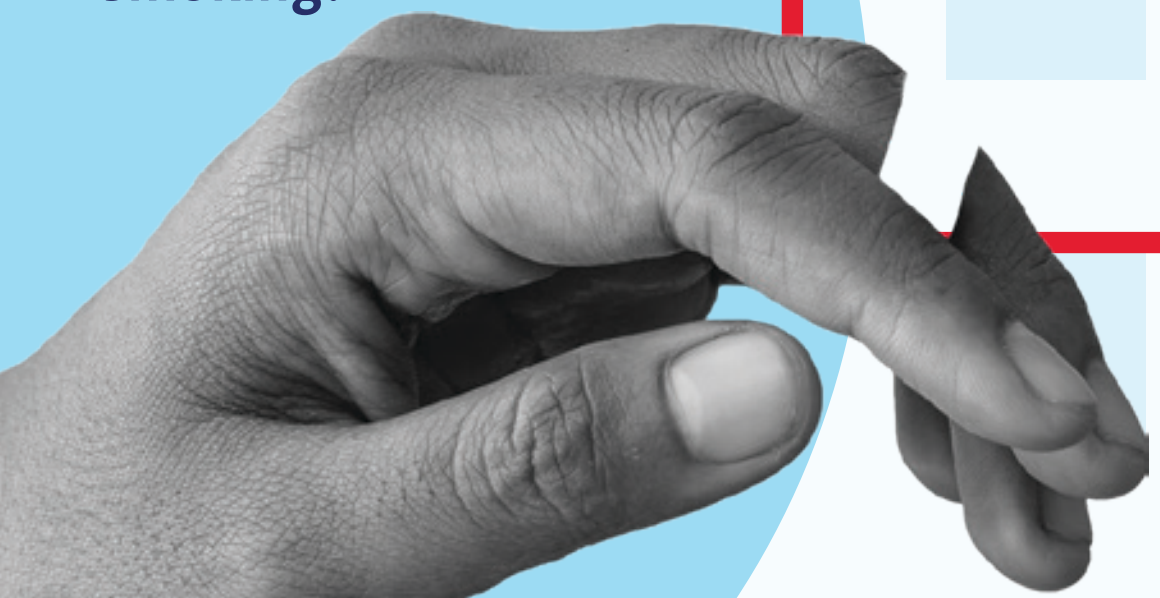

Quitting smoking is hard because nicotine is highly addictive. Many parents want to quit but often struggle to do so without support.

Design: Liesbeth Smit, The Online Scientist

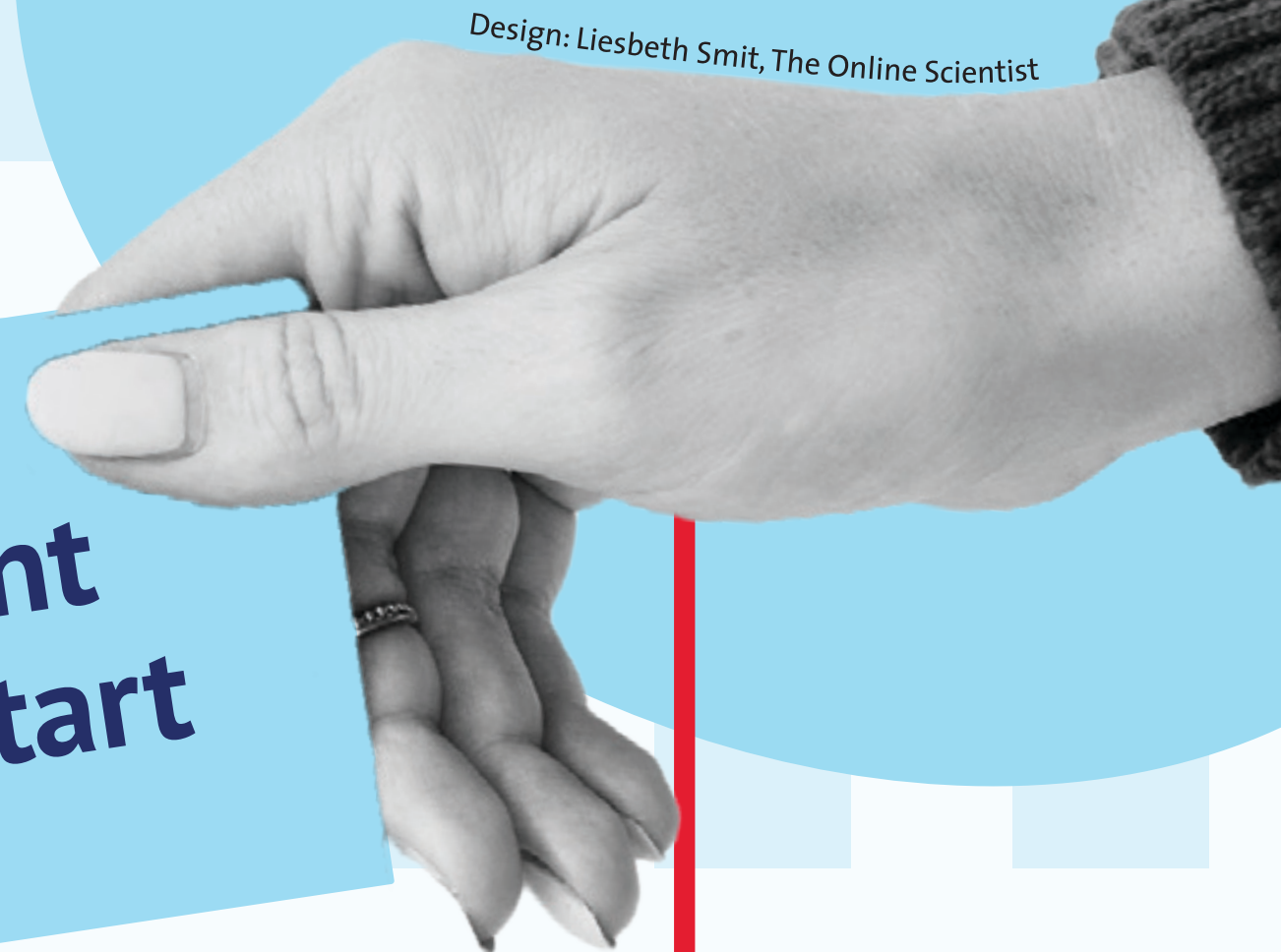

A smart investment in a smoke-free start

With vouchers as an addition to existing coaching programmes, parents are two to three times more likely to quit successfully compared with coaching alone.

Lower healthcare costs

Health gains

More children with a healthy start in life

This presents an opportunity for the Netherlands. By making a small addition to existing coaching programmes, we can help more parents quit smoking and improve the health of future generations.

In England, vouchers are already part of standard care for pregnant women who stop smoking.

Learn more about this project: www.rookvrijestart.nl/onderzoek
Contact: Dr. Jasper Been j.been@erasmusmc.nl

Erasmus MC Sophia Kinderziekenhuis

rookvrije start

Trimbos instituut

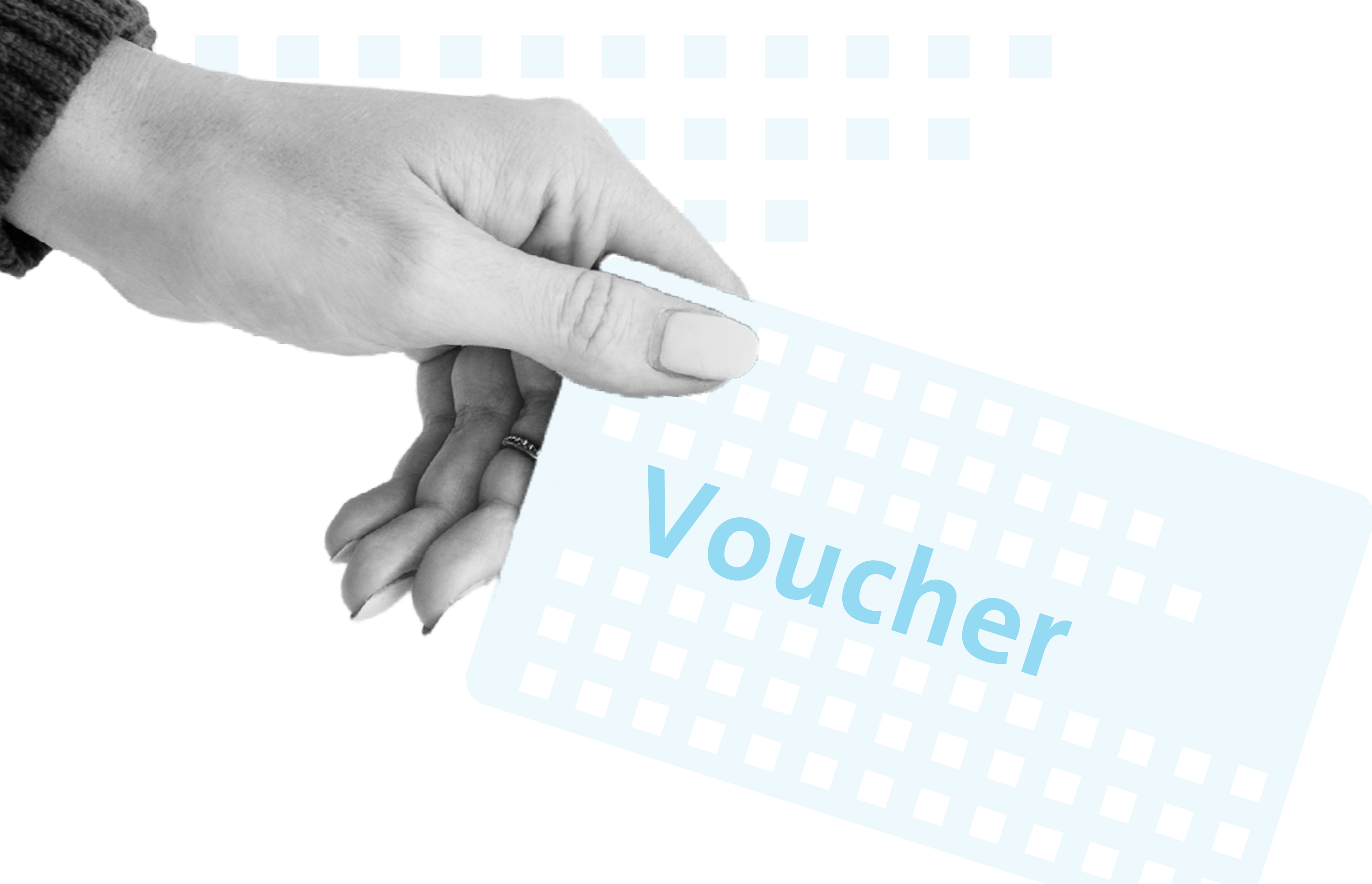

This policy brief outlines how this evidence-based approach can be implemented in the Netherlands.

It is based on research conducted with policymakers, health insurers, end-users, and other stakeholders, who together explored what a voucher-based programme could look like, which challenges and opportunities exist, and what recommendations can be made for successful implementation.

Jasper Been, MD MPH PhD

Paediatrician-neonatologist,
Erasmus MC Sophia Children's Hospital

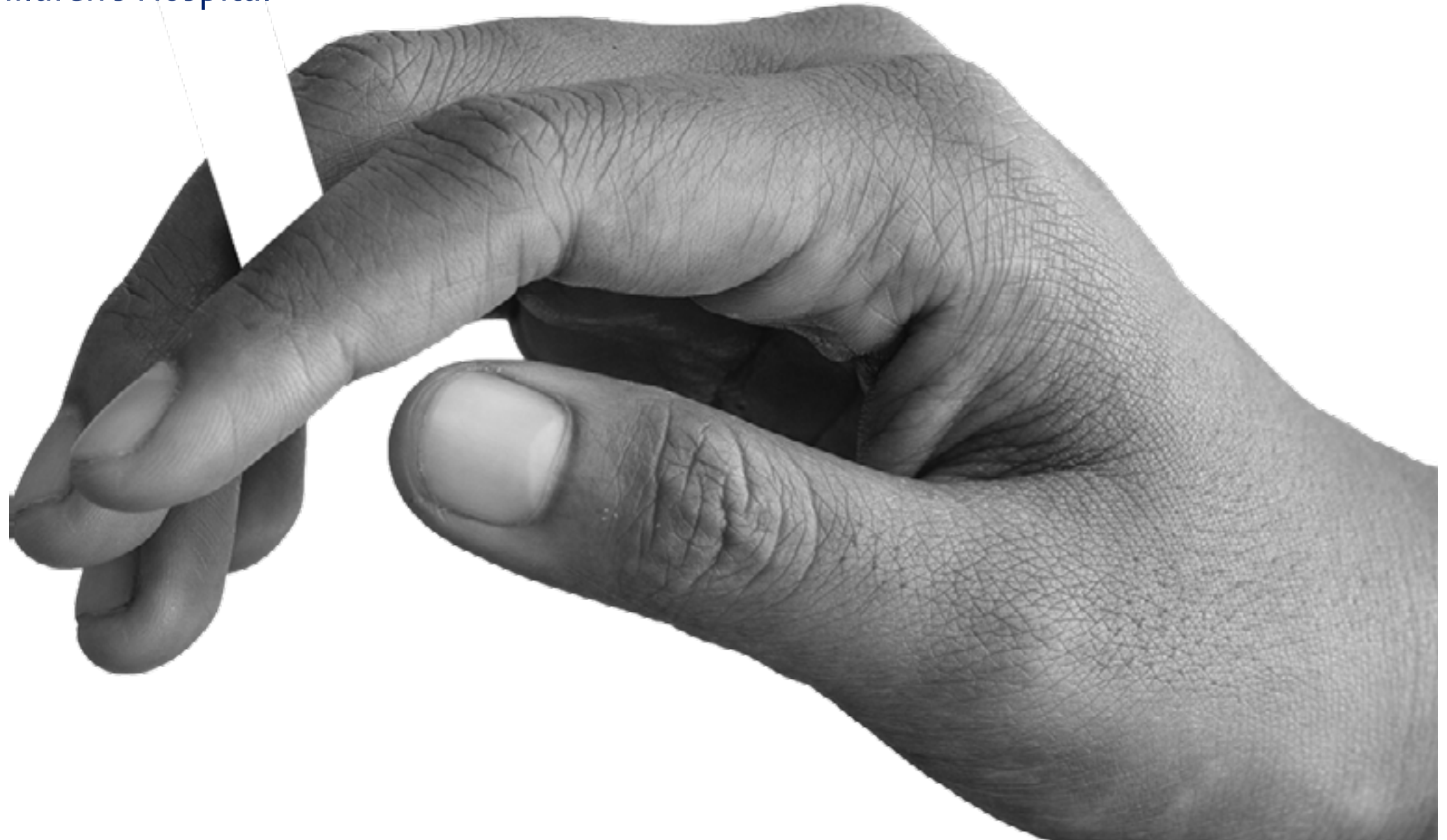

POLICY BRIEF

Investing in a Smoke-Free Start

Vouchers for (expectant) parents who quit smoking

Problem

Children are exposed to harmful tobacco smoke

Every day in the Netherlands, 28 babies enter the world already affected by tobacco smoke before birth – a harm they neither chose nor can prevent. For many (expectant) parents, however, quitting is extremely difficult: smoking is a serious addiction.

Solution

Coaching with vouchers supports parents to quit

Coaching can make a major difference. Telephone-based programmes such as Smoke-free Parents (Rookvrije Ouders) are proven effective and freely available. Still, the threshold to seek help remains high, and not all parents who want to quit manage to access such support.

Financial incentives in the form of vouchers can make coaching both more attractive and more effective. They double the likelihood of quitting success, improve the health of both parent and child, and reduce healthcare costs.

Watch our explainer video about vouchers at rookvrijestart.nl/onderzoek

Smoking during pregnancy

In the Netherlands, **13%** of women smoke just before pregnancy, **6%** during pregnancy, and **7%** after pregnancy. Each year, more than 10,000 pregnant women smoke[1].

A quarter of all partners of pregnant women smoke, and among women who smoke, over three quarters have a partner who also smokes. In total, more than one million parents in the Netherlands smoke[1-2].

Moreover, inequalities are widening: women with a lower level of education are much more likely to smoke during pregnancy than those with higher education levels[1,3].

6% of women smoke during pregnancy

10,500 women each year

In total, more than **1 million** parents smoke

24% of partners of pregnant women and women planning a pregnancy smoke.

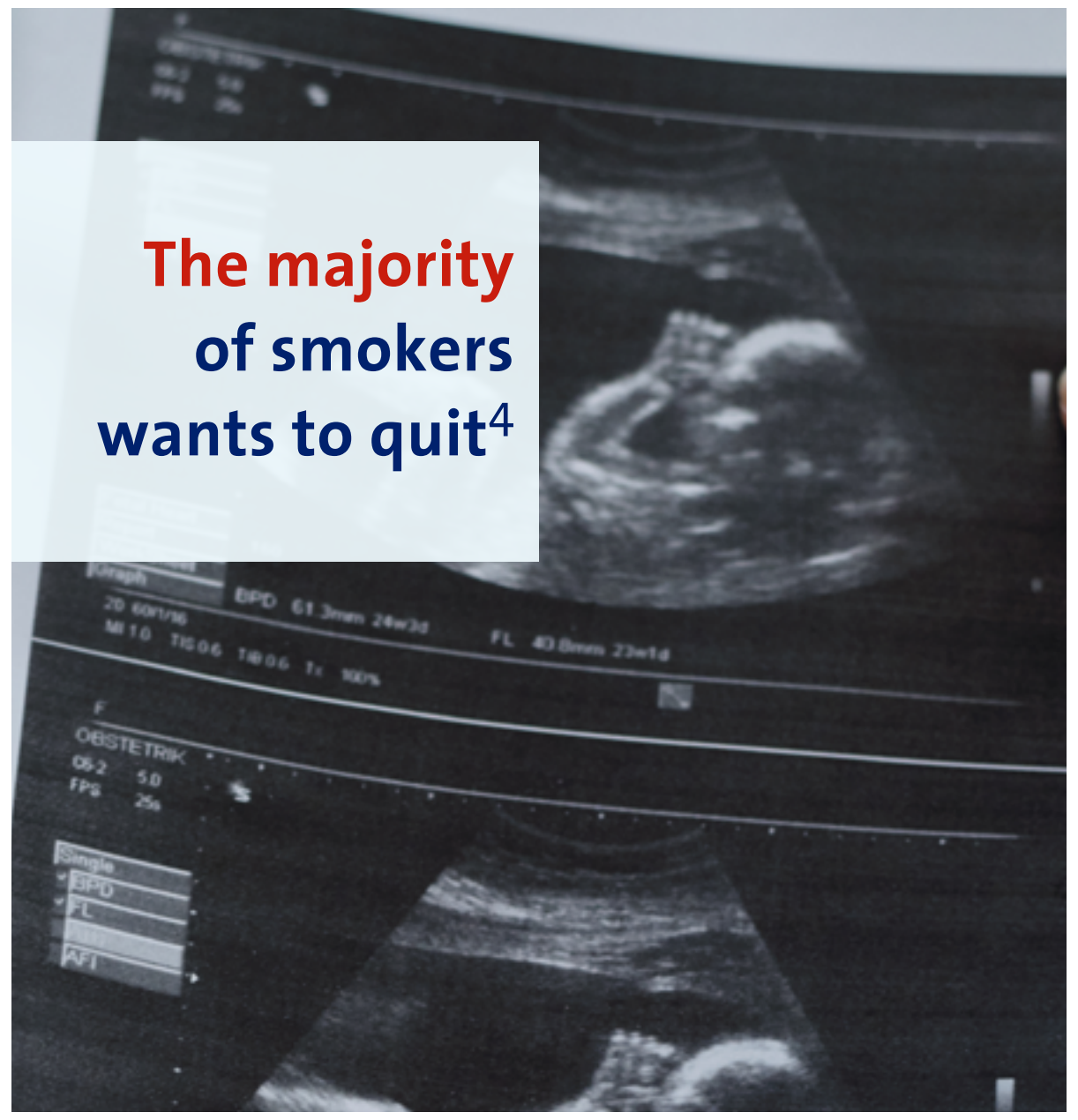

The majority of smokers wants to quit[4]

Smoking during pregnancy is marked by inequality:

20.3%	Basic/vocational (vmbo)
9.9%	Secondary (havo/vwo/mbo)
2.5%	Higher (hbo/wo)

Risks of smoking

Smoking is the number one preventable cause of illness in the Netherlands, leading to serious health risks and high healthcare costs, particularly around pregnancy.

Smoking during pregnancy increases the risk of:

- cancer
- miscarriage
- COPD
- asthma
- low birth weight
- a child taking up smoking
- cardiovascular disease
- premature birth

Burden of disease

7.6%

of the national disease burden is attributable to smoking[3].

Healthcare costs

€2.4 billion

of total healthcare costs are attributable to smoking[5].

Smoking is the leading preventable cause of disease in the Netherlands and contributes substantially to the national disease burden and healthcare costs. In addition to illness and mortality, smoking causes major societal costs due to reduced productivity and diminished quality of life.

The National Prevention Agreement aims for no smoking during pregnancy by 2040. It is more urgent than ever to invest in cost-effective measures such as vouchers, as part of broader efforts to address smoking and its underlying causes.

"By 2040, no pregnant woman will smoke"

Goal of the Dutch National Prevention Agreement

Coaching with vouchers: it’s effective

Vouchers make parents 2 to 3 times more likely to quit smoking

It’s the most effective method

(Expectant) parents who receive vouchers are two to three times more likely to quit smoking than those who receive coaching alone. They are also more likely to remain smoke-free in the long term[6-9].

There are only costs if someone successfully quits. In other words: no cure, no pay.

It improves health and saves money

Vouchers are not only cost-effective but even cost-saving[10-11]. Quitting smoking reduces the likelihood of complications such as premature birth, and lowers healthcare costs by preventing smoking-related illness.

It contributes to a solid start

Investing in cessation programmes contributes to a healthier start in life and helps reduce health inequalities.

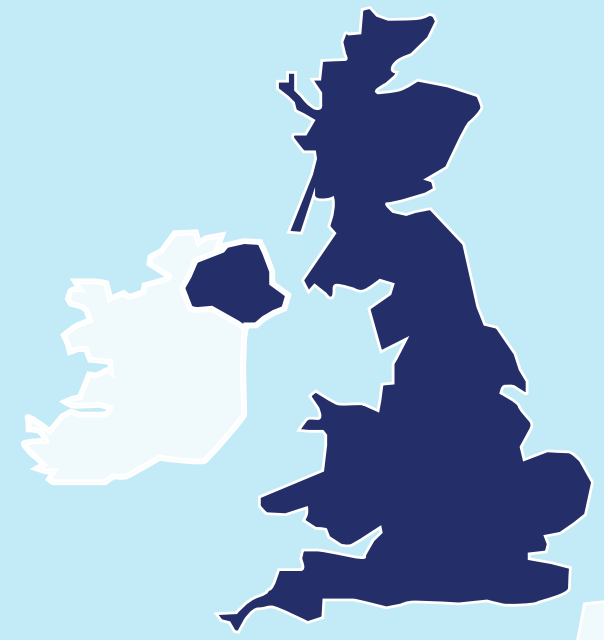

In the United Kingdom, it has been shown that vouchers lead to long-term savings in healthcare costs for both mother and child, as well as improved quality of life[11].

In the largest part of England, vouchers are already part of routine care for pregnant women who quit smoking and are included in NICE guidelines as a cost-effective form of care.

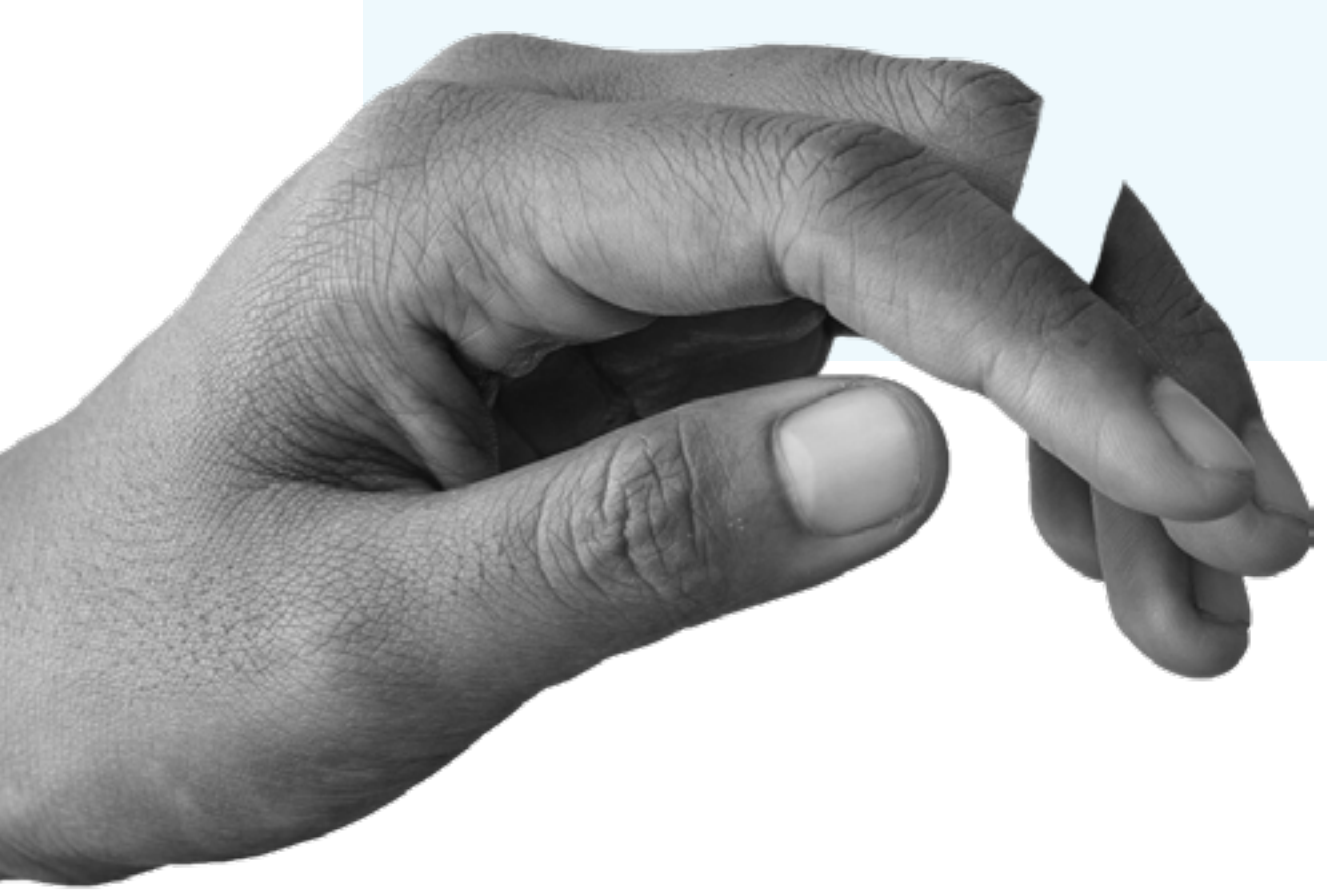

Parents who receive vouchers are more likely to quit smoking and remain smoke-free.

	Smoke-free after pregnancy		Smoke-free in the long-term[9]
	25% smoke-free	with vouchers	13% smoke-free
	9% smoke-free	without vouchers	6% smoke-free

*These percentages refer to pregnant women receiving coaching, not to those who quit without support.

Relapse

Relapse unfortunately occurs frequently, but even a smoke-free pregnancy already provides substantial health benefits for the baby.
By rewarding verified abstinence at multiple stages, vouchers support sustained engagement with cessation support and reduce the likelihood of relapse.

How it works

1 Referral

Healthcare professionals discuss smoking cessation with (expectant) parents and refer them to a coaching programme with vouchers. Parents can also register online.

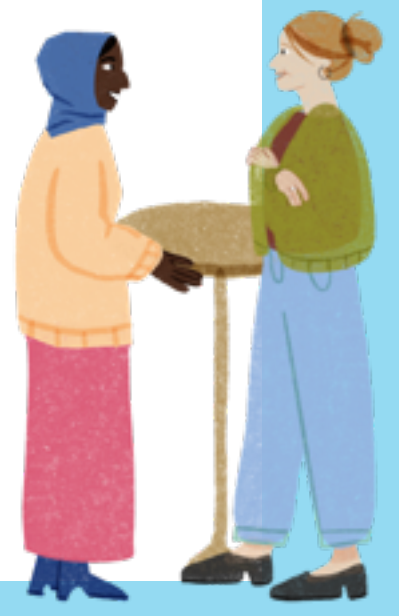

2 Coaching

Participants are counselled by a professional smoking cessation coach.

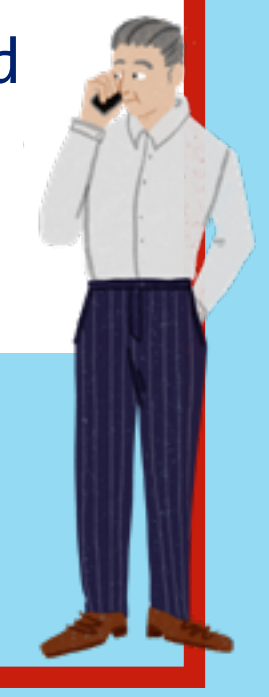

3 The participants quits smoking

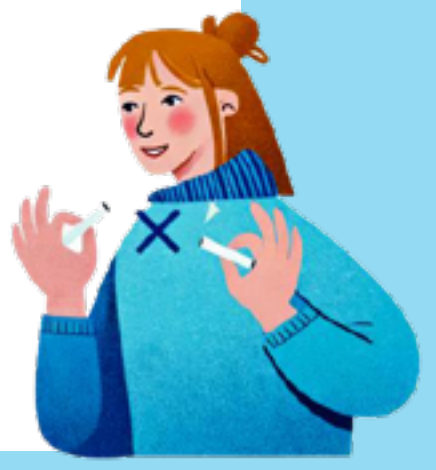

Vouchers are not a quick fix, but an effective tool for parents who already want to quit.

4 Breath test

At each milestone, such as one week or one month smoke-free, the participant takes a carbon monoxide breath test.

This test prevents gaming and is often experienced as motivating.

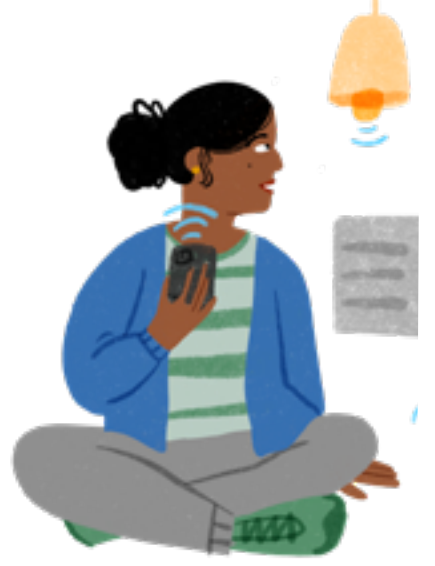

5 Smoke-free? Voucher!

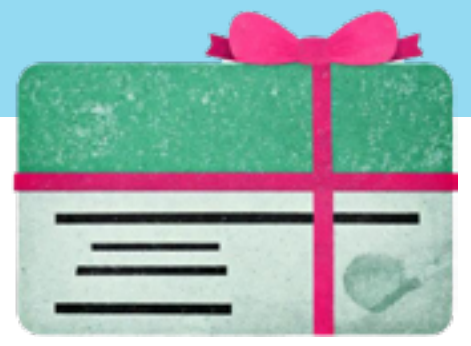

At every verified smoke-free milestone, the participant receives a voucher.
Vouchers replace, in part, the reward that nicotine normally gives to the brain, helping participants cope with withdrawal symptoms and remain motivated.

From scepticism to support:

Success stories strengthen support

While not everyone initially agrees with rewarding parents for quitting smoking, public support grows as people see the results.

About one in three Dutch citizens view vouchers for pregnant women who quit smoking as a good idea[12].

Both information campaigns and pilot projects can increase awareness and support among a wider audience.

After watching a short explanatory video (see page 3), support for vouchers doubled to over 60%[13].

Also, people directly involved in these programmes (e.g., participants and healthcare professionals) often become more positive once they see how well it works[14].

The greater the support, the greater the feasibility of sustainable funding.

Common misconceptions

- "Isn't quitting smoking a personal responsibility?" Addiction, stress, and social environment all play a major role. Just like with other chronic conditions or addictions, support is essential.
- "Do parents start smoking just to receive vouchers?" Evidence shows they don't[9].
- "Do vouchers undermine intrinsic motivation?" No, they actually help strengthen confidence and motivation to quit.
- "Is it unfair that smokers receive vouchers while non-smokers get nothing?" Ultimately, everyone benefits from healthier generations and lower healthcare costs.

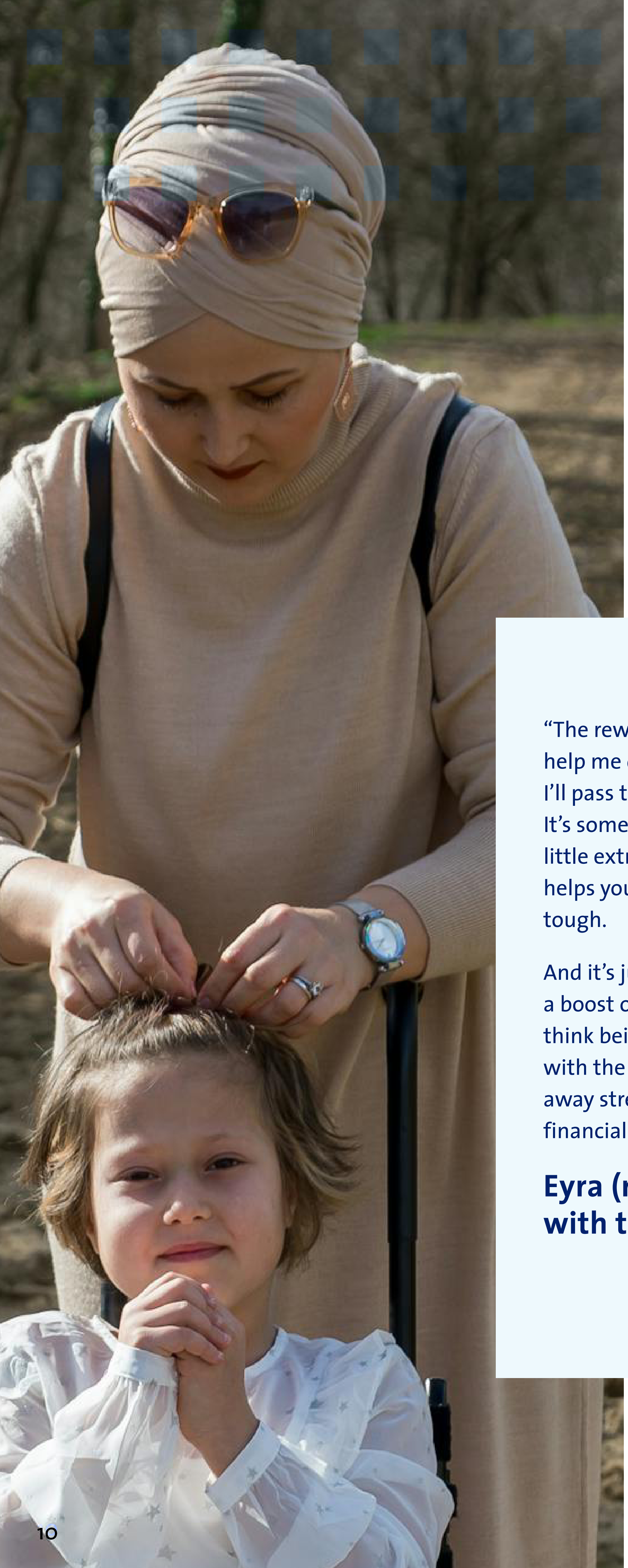

Investment in voucher-based programmes leads to health gains across the life course of mother and child, cost savings, and reduced health inequalities.

"The reward was more like an extra push to help me quit. I'd think: 'if I don't smoke now, I'll pass the breath test at the next session...' It's something you can tell yourself as a little extra motivation, something that helps you correct yourself when things get tough.

And it's just a bright spot, too. You get a boost of joy every time it works. And I think being able to buy things for your kids with the vouchers is helpful. It also takes away stress for people who are struggling financially."

Eyra (mother) quit smoking with the help of vouchers

Recommendations

Practical tips for implementation

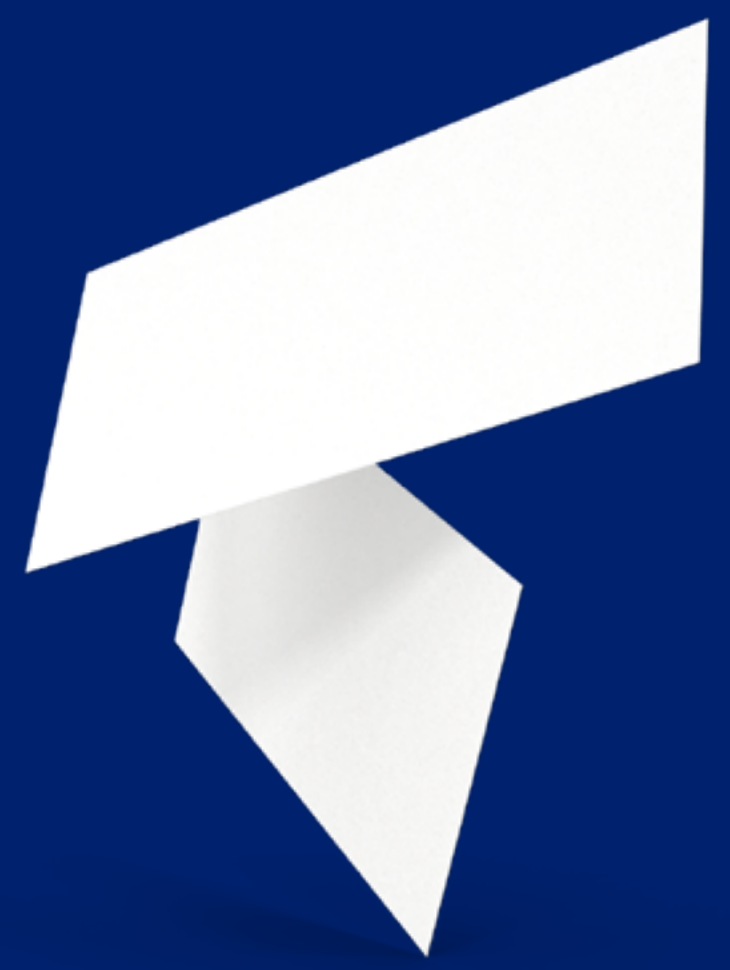

Ensure accessibility

Add vouchers to existing and well-known programmes (such as Smoke-free Parents). The breath test should be easy to perform—either at home (digitally) or at a familiar setting such as the GP, midwife or child health clinic.

Together is better

Encourage participation with the partner to increase reach and improve outcomes.

Keep it simple

The programme should be clear and straightforward, without complicated schemes or procedures. It must remain accessible for people with lower health literacy or limited financial means. For healthcare professionals as well, offering vouchers should not be time-consuming.

Involve everyone

Engage healthcare professionals, coaches and participants in the design process to ensure successful implementation

Reward immediately

Provide the voucher directly after the breath test – this has the strongest impact.

Make it worthwhile

A total value of around €400 spread over six months has proven effective. Higher or lower amounts may also work, depending on the context.

Make it relevant

A (digital) voucher for groceries, baby products or other everyday items is practical, motivating and contributes to the wellbeing of both parent and child.

A broadly usable voucher meets personal needs and motivates more people to take part, while a focus on health-related products can help strengthen public support.

Dutch examples

SAFER Pregnancy Study[16]

What? Group coaching, online platform and vouchers (max. €185)

Who? 39 participants (pregnant or planning pregnancy)

RESULT **28% quit smoking,**
high satisfaction, though recruitment proved challenging.

Samen Sterker Stoppen[17]

What? Group coaching and vouchers (max. €350)

Who? In 61 companies, with 604 employees and partners

RESULT **41% smoke-free after 12 months,**
compared to 26% without vouchers.

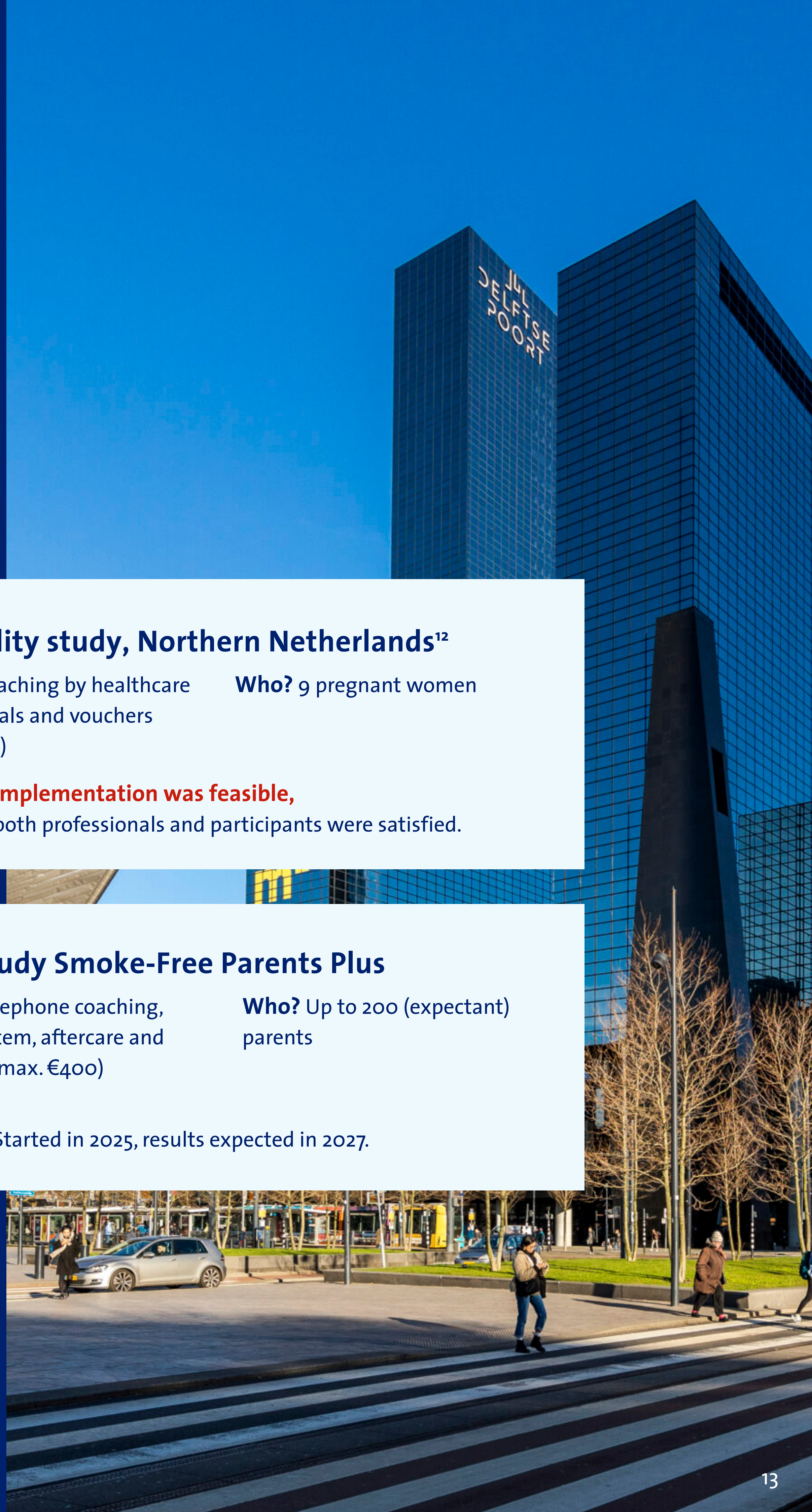

Feasibility study, Northern Netherlands[12]

What? Coaching by healthcare professionals and vouchers (max. €575)

Who? 9 pregnant women

RESULT **Implementation was feasible,**
both professionals and participants were satisfied.

Pilot study Smoke-Free Parents Plus

What? Telephone coaching, buddy system, aftercare and vouchers (max. €400)

Who? Up to 200 (expectant) parents

STATUS Started in 2025, results expected in 2027.

Investing in a cost-effective addition to care

The main challenge for health insurers is to identify a feasible way to finance vouchers. Smoking cessation support is already covered by the basic health insurance package, but additional incentives such as vouchers are not (yet) included. Under the Dutch Health Insurance Act, vouchers fall outside the legal definition of healthcare, and selective targeting of (expectant) parents is not permitted. For these reasons, vouchers are not covered by basic health insurance. Nevertheless, vouchers provide a proven, accessible and cost-effective way to increase the success rate of smoking cessation programmes.

Start with a pilot

A small-scale pilot can generate valuable insight into the health gains and potential cost savings that this innovative addition can deliver. By temporarily funding vouchers from their own resources, insurers can experiment, gather evidence, and evaluate the impact—possibly with a cap on participant numbers, through partnerships, or via a supplementary insurance package. A successful pilot can provide the basis for broader scale-up and for discussions with policymakers on structural funding.

Ensure equal access

Vouchers are most effective when they are accessible to all participants, including those without supplementary insurance. Excluding vulnerable groups (where health gains are often greatest) should be avoided. Indirect incentives, such as premium discounts, are not recommended, as they tend to be less effective in changing behaviour.

An investment in health and reputation

Investing in smoking cessation first and foremost improves health outcomes and quality of life, while also reducing healthcare expenditure in the long term. At the same time, supporting evidence-based prevention programmes enables insurers to position themselves as leaders in preventive care.

From pilots to structural implementation

Integrate into existing care

Integrating vouchers into existing, recognised smoking cessation programmes already reimbursed under the basic health insurance package eliminates the need for new delivery structures and increases both reach and effectiveness.

Bring relevant stakeholders together

Facilitate collaboration between the Ministry of Health, the National Health Care Institute (Zorginstituut Nederland), the Dutch Healthcare Authority (NZa), and health insurers to remove legal and financial barriers and define the conditions for implementation.

One key obstacle is that financial incentives are currently excluded from the legal definition of healthcare and therefore from the basic insurance package. This is inconsistent with the growing understanding that effective prevention extends beyond medical treatment alone, and warrants further consideration and exploration by relevant stakeholders.

Explore funding options

In addition to reimbursement through basic health insurance, alternative funding routes could be explored, such as: (a) introducing a levy on the tobacco industry to create a prevention fund (as in Canada); (b) allocating tobacco tax revenues to smoking prevention; or (c) local funding through municipal initiatives such as Kansrijke Start (Solid Start). Local financing, however, may result in regional inequalities, underlining the importance of a coordinated national approach.

Support pilot projects

Encourage and fund pilot projects to enable national scale-up and to stimulate public discussion about the legal and financial preconditions. Clear evidence of real-world impact can help build support for policy change.

About this research

This research is supported by Smarter Choices for Better Health and Convergence | Healthy Start, a program of the Convergence Alliance – Delft University of Technology, Erasmus University Rotterdam, and Erasmus MC – to improve the future of new generations.

Convergence Healthy Start

Acknowledgements

This policy brief was developed based on insights from a participatory Policy Lab involving professionals, policymakers, people with lived experience, and researchers from: the Ministry of Health, Welfare and Sport; the National Health Care Institute; Radboud University; Maastricht University; Coöperatie VGZ; Zilveren Kruis; MediPro; the Smoke-free Start Taskforce; Erasmus MC Sophia Children's Hospital; the Partnership Stop Smoking; and the Dutch Healthcare Authority.

Watch our explainer video about vouchers at rookvrijestart.nl/onderzoek

Erasmus MC Sophia Kinderziekenhuis

Trimbos instituut

Erasmus School of Economics

rookvrije start

TU Delft

Onderzoekers

Linda van der Spek
Erasmus MC Sophia Children's Hospital and Trimbos Institute
lspek@trimbos.nl

Leonieke Breunis
Erasmus MC Sophia Children's Hospital
l.breunis@erasmusmc.nl

Niek Mouter
Delft University of Technology
n.mouter@tudelft.nl

Tessa Scheffers-van Schayck
Trimbos Institute
tscheffers@trimbos.nl

Hans van Kippersluis
Erasmus School of Economics
hvankippersluis@ese.eur.nl

Jasper Been
Erasmus MC Sophia Children's Hospital
j.been@erasmusmc.nl

Design by Liesbeth Smit, The Online Scientist

References

1. Scheffers-van Schayck T, Ramaker V, Van Engen M, Morren K, Tuithof M. Monitor Middelengebruik en Zwangerschap 2023. [Substance Use and Pregnancy Monitor 2023]. Utrecht: Trimbos Institute; 2024. https://www.trimbos.nl/aanbod/ webwinkel/af2186-monitormiddelengebruik-en-zwangerschap-2023/

2. Van Westen-Lagerweij N, Van den Eeden N, Bommelé J. Meeroken door kinderen. Feiten en cijfers 2020. [Second-hand Smoke Exposure among Children: Facts and Figures 2020]. Utrecht: Trimbos Institute; 2022. https://www.trimbos.nl/wp content/uploads/2022/05/INF119-Meerokendoor-kinderen.pdf

3. RIVM (National Institute for Public Health and the Environment). Volksgezondheid Toekomst Verkenning 2024 [Public Health Foresight Study 2024]. Bilthoven: RIVM; 2024. https://www.olksgezondheidtoekomstverkenning.nl/vtv-2024/hoofdrapport

4. Aanvullende Module Middelen van de Leefstijlmonitor (LSM-A middelen) [Supplementary Module on Substance Use from the Lifestyle Monitor]. Trimbos-instituut i.s.m. RIVM en CBS, 2020.

5. RIVM (National Institute for Public Health and the Environment). Volksgezondheid Toekomst Verkenning 2018 [Public Health Foresight Study 2018]. Bilthoven: RIVM; 2018. https://www.rivm.nl/publicaties/ volksgezondheid-toekomst-verkenning-2018-gezond-vooruitzicht-synthese

6. Kock LS, Erath TG, Coleman SRM, Higgins ST, Heil SH. Contingency management interventions for abstinence from cigarette smoking in pregnancy and postpartum: A systematic review and meta-analysis.Prev Med. 2023;(July):107654. doi:10.1016/j.ypmed.2023.107654

7. Vila-Farinas A, Perez-Rios M, Montes-Martinez A, et al. Effectiveness of smoking cessation interventions among pregnant women: An updated systematic review and meta analysis. Addict Behav. 2024;148(August 2023). doi:10.1016/j.addbeh.2023.107854

8. Chamberlain C, O'Mara-Eves A, Porter J, et al. Psychosocial interventions for supporting women to stop smoking in pregnancy. Cochrane Database Syst Rev. 2017;2017(2). doi:10.1002/14651858.CD001055.pub5

9. Notley C, Gentry S, Livingstone-Banks J, Bauld L, Perera R, Hartmann-Boyce J. Incentives for smoking cessation. Cochrane Database Syst Rev. 2025. doi:10.1002/14651858.CD004307.pub7

10. Mundt MP, Baker TB, Piper ME, Smith SS, Fraser DL, Fiore MC. Financial incentives to Medicaid smokers for engaging tobacco quit line treatment: Maximising return on investment. Tob Control. 2020;29(3):320-325. doi:10.1136/tobaccocontrol-2018-054811

11. McMeekin N, Sinclair L, Robinson-Smith L, et al. Financial incentives for quitting smoking in pregnancy: Are they cost-effective? Addiction. 2023;(February):1-12. doi:10.1111/add.16176

12. Authors' own calculations based on Kantar data and LISS panel (forthcoming)

13. Authors' own calculations based on LISS panel (forthcoming)

14. Kroder TA, Peters LL, Roggeveld AL, et al. Smoking cessation in pregnant women using financial incentives: a feasibility study. BMC Pregnancy Childbirth. 2022;22(1):1-14. doi:10.1186/s12884-022-05292-9

15. Breunis LJ, de Kroon MLA, de Jong-Potjer LC, Steegers EAP, Been J V. Piloting a complex intervention to promote a tobacco and alcohol-free pregnancy: the Smoke and Alcohol Free with EHealth and Rewards (SAFER) pregnancy study. BMC Pregnancy Childbirth. 2023;23(1):1-14. doi:10.1186/s12884-022-05320-8

16. Van den Brand FA, Nagelhout GE, Winkens B, Chavannes NH, van Schayck OCP. Effect of aworkplace based group training programme combined with financial incentives on smoking cessation: acluster randomised controlled trial. Lancet Public Heal. 2018;3(11):e536-e544. doi:10.1016/S2468-2667(18)30185-3
