# Supplementary material for: Stakeholder Perspectives on National Implementation of Financial Incentives for Smoking Cessation Before, During and After Pregnancy: A Policy Lab Study
Source: Health Expect. 2026 Oct 12;29(5):e70893. doi: 10.1111/hex.70893 (PMC13656341; doi:10.1111/hex.70893)
Supplement: Supplementary file 4 [file HEX-29-e70893-s002.docx]

#### Supplementary File 5: Baseline questionnaire results

| **Perceived effectiveness**: To what extent do you think it is effective to support (expectant) parents in quitting smoking by providing incentives? | | |
| --- | --- | --- |
| **Response** | **N** | **Comments** |
| Not convinced | 1 |  |
| Neutral | 2 | “I wonder how sustainable quitting is if the required motivation is partly based on a reward.” “In my opinion, this could be of added value if it is known for whom this does or does not work. What are the characteristics of people for whom this does or does not work and which incentives are most effective in this. The moment a distinction is made in this, there is unequal treatment. Also an ethical issue. For whom is an incentive and for whom is it not?” |
| Convinced | 6 | “Experienced it myself.” “I definitely think that giving rewards can help promote healthy behaviors. I do think that it is also very important that parents, for example, also receive good support in quitting smoking. So that the reward is not the only factor that helps.” |
| **Support** for government funding: To what extent do you think it is desirable for the government to support (expectant) parents in quitting smoking by providing rewards? | | |
| **Response** | **N** | **Comments** |
| Not desirable | 1 | “Although I would like everyone to stop smoking, I wonder if rewards are the right step. It sets a precedent for other addictions or lifestyle goals that we want to achieve. Why reward quitting smoking, and not losing weight, exercise etc?”  “I also wonder whether this is an effectual use of resources. Could the money not be better directed towards other necessary care?” |
| Neutral | 4 | “For now, I filled in neutrally. Depending on the costs and benefits in the short and long term (within a year and longer than a year) and also on how this issue is viewed ethically, this may be desirable. Smoking cessation care is reimbursed from the basic insurance and is exempt from deductible. Nationally, the reimbursements within the basic insurance are the same for all health insurers, so this should therefore be at the level of, among others, the NZA (*National Health Care Institute*) and ZN (*Dutch Association of Health insurers*).”  “I do think that the government can play a role, but right now I don't know in what way/in what form. It may also be difficult that this is a very specific target group.” |
| Desirable | 4 | “We need to deploy the most effective and cost-effective intervention.” |
| **Support** for insurer funding: To what extent is it desirable for health insurers to offer rewards to (expectant) parents who quit smoking? | | |
| **Response** | **N** | **Comments** |
| Not desirable | 2 | “Tax conditions challenges: insurance activities are exempt from VAT. If incentives are given away under the insurance contract, this can be said to relate directly to our exempt insurance activities. What does this do to the affordability of the premium and for whom is an incentive used or not?” |
| Neutral | 3 | “Government or health insurers does not matter. Unless it would be paid for from the supplementary package.” “I do think that a health insurer can support this, also because various insurers have already had programs in the past to achieve behavioral change with rewards. However, it may also be the case here that the target group is a bit more complicated (than, for example, exercise behavior) (how do you reach these people and make them want to make the change themselves)” |
| Desirable | 4 |  |

*Results of the baseline questionnaire, assessing perspectives on perceived effectiveness and desirability, completed by nine of twelve participants.*
