# Supplementary material for: Stakeholder Perspectives on National Implementation of Financial Incentives for Smoking Cessation Before, During and After Pregnancy: A Policy Lab Study
Source: Health Expect. 2026 Oct 12;29(5):e70893. doi: 10.1111/hex.70893 (PMC13656341; doi:10.1111/hex.70893)
Supplement: Supplementary file 5 [file HEX-29-e70893-s004.docx]

#### Supplementary File 4: Codebook

| **Code** | **Definition** |
| --- | --- |
| **I. INNOVATION DOMAIN** | **Innovation: Financial incentives for smoking cessation among (expectant) parents (The “thing” being implemented)** |
| A. Innovation Source | The degree to which the group that developed and/or visibly sponsored use of the innovation is reputable, credible, and/or trustable. |
| B. Innovation Evidence-Base | The degree to which the innovation has robust evidence supporting its effectiveness. |
| C. Innovation Relative Advantage | The degree to which the innovation is better than other available innovations or current practice. |
| D. Innovation Adaptability | The degree to which the innovation can be modified, tailored, or refined to fit local context or needs. |
| E. Innovation Trialability | The degree to which the innovation can be tested or piloted on a small scale and undone. |
| F. Innovation Complexity | The degree to which the innovation is complicated, which may be reflected by its scope and/or the nature and number of connections and steps. |
| 1. Integration in existing program (+) | The degree to which the innovation can be integrated in an existing program, which may be reflected by less complexity |
| G. Innovation Design | The degree to which the innovation is well designed and packaged, including how it is assembled, bundled, and presented. |
| 1. Selective targeting (+) | The degree to which a specific population is targeted with the innovation. |
| 2. Coaching program design and quality (+) | The design and quality of the coaching program in which incentives are embedded. |
| 3. Form of incentive (+) | The incentive form, e.g., vouchers, cash or discount. |
| 4. Gaming and biochemical validation (+) | Biochemical validation procedures and prevention of gaming. |
| H. Innovation Cost | The degree to which the innovation purchase and operating costs are affordable. |
| 1. Insight and fidelity in cost-effectiveness (+) | The degree to which there is insight and fidelity in cost-effectiveness and preferably short-term benefits |
| **II. IMPLEMENTATION SETTING DOMAIN** | **Dutch smoking cessation support policy and practice (the setting in which the innovation is implemented).** |
| A. Critical Incidents | The degree to which to large-scale and/or unanticipated events disrupt implementation and/or delivery of the innovation. |
| B. Local Attitudes | The degree to which sociocultural values (e.g., shared responsibility in helping recipients) and beliefs (e.g., convictions about the worthiness of recipients) encourage the Outer Setting to support implementation and/or delivery of the innovation. |
| C. Local Conditions | The degree to which economic, environmental, political, and/or technological conditions enable the Outer Setting to support implementation and/or delivery of the innovation. |
| D. Partnerships & Connections | The degree to which the Inner Setting is networked with external entities, including referral networks, academic affiliations, and professional organization networks. |
| E. Policies & Laws | The degree to which legislation, regulations, professional group guidelines and recommendations, or accreditation standards support implementation and/or delivery of the innovation. |
| F. Financing | The degree to which funding from external entities (e.g., grants, reimbursement) is available to implement and/or deliver the innovation. |
| 1. Financing from insurers’ own budgets or supplementary insurance (+) | Funded from health insurers' own funds or from supplementary insurance. |
| 2. Basic insurance (+) | Reimbursed as part of the basic insurance package. |
| G. External Pressure | The degree to which external pressures drive implementation and/or delivery of the innovation. Note: Use this construct to capture themes related to External Pressures that are not included in the subconstructs below. |
| 1. Societal Pressure | The degree to which mass media campaigns, advocacy groups, or social movements or protests drive implementation and/or delivery of the innovation. |
| 2. Market Pressure | The degree to which competing with and/or imitating peer entities drives implementation and/or delivery of the innovation. |
| 3. Performance-Measurement Pressure | The degree to which quality or benchmarking metrics or established service goals drive implementation and/or delivery of the innovation. |
| A. Structural Characteristics | The degree to which infrastructure components support functional performance of the Inner Setting. Subconstructs: Space/tangible/information technology/staffing |
| B. Relational Connections | The degree to which there are high quality formal and informal relationships, networks, and teams within and across Inner Setting boundaries (e.g., structural, professional). |
| C. Communications | The degree to which there are high quality formal and informal information sharing practices within and across Inner Setting boundaries (e.g., structural, professional). |
| D. Culture | The degree to which there are shared values, beliefs, and norms across the Inner Setting. Note: Use this construct to capture themes related to Culture that are not included in the subconstructs below. |
| 1. Human Equality-Centredness | The degree to which there are shared values, beliefs, and norms about the inherent equal worth and value of all human beings. |
| 2. Recipient-Centredness | The degree to which there are shared values, beliefs, and norms around caring, supporting, and addressing the needs and welfare of recipients. |
| 3. Deliverer-Centredness | The degree to which there are shared values, beliefs, and norms around caring, supporting, and addressing the needs and welfare of deliverers. |
| 4. Learning-Centredness | The degree to which here are shared values, beliefs, and norms around psychological safety, continual improvement, and using data to inform practice. |
| E. Tension for Change | The degree to which the current situation is intolerable and needs to change. |
| F. Compatibility | The degree to which the innovation fits with workflows, systems, and processes. |
| G. Relative Priority | The degree to which implementing and delivering the innovation is important compared to other initiatives. |
| H. Incentive Systems | The degree to which tangible and/or intangible incentives and rewards and/or disincentives and punishments support implementation and delivery of the innovation. |
| I. Mission Alignment | The degree to which implementing and delivering the innovation is in line with the overarching commitment, purpose, or goals in the Inner Setting. |
| J. Available Resources | The degree to which resources are available to implement and deliver the innovation |
| 1. Funding | The degree to which funding is available to implement and deliver the innovation. |
| 2. Space | The degree to which physical space is available to implement and deliver the innovation. |
| 3. Materials & Equipment | The degree to which supplies are available to implement and deliver the innovation. |
| K. Access to Knowledge & Information | The degree to which guidance and/or training is accessible to implement and deliver the innovation. |
| **IV. INDIVIDUALS DOMAIN** | **Individuals: The roles and characteristics of individuals.** |
| A. High-level Leaders: Politicians | Individuals with a high level of authority, including key decision-makers, executive leaders, or directors. |
| 1. Need | The degree to which high-level leader(s) have deficits related to survival, well-being, or personal fulfillment, which will be addressed by implementation and/or delivery of the innovation. |
| 2. Capability | The degree to which high-level leader(s) have interpersonal competence, knowledge, and skills to fulfill Role. |
| 3. Opportunity | The degree to which high-level leader(s) have availability, scope, and power to fulfill Role. |
| 4. Motivation | The degree to which high-level leader(s) are committed to fulfilling Role. |
| B. Mid-level Leaders: Policymakers (e.g., National Healthcare Institute (Zi), Dutch Healthcare Authority (NZa), Ministry of Health) | Individuals with a moderate level of authority, including leaders supervised by a high-level leader and who supervise others. |
| 1. Need | The degree to which mid-level leader(s) have deficits related to survival, well-being, or personal fulfillment, which will be addressed by implementation and/or delivery of the innovation. |
| 2. Capability | The degree to which mid-level leader(s) have interpersonal competence, knowledge, and skills to fulfill Role. |
| 3. Opportunity | The degree to which mid-level leader(s) have availability, scope, and power to fulfill Role. |
| 4. Motivation | The degree to which mid-level leader(s) are committed to fulfilling Role. |
| C. Opinion Leaders: | Individuals with informal influence on the attitudes and behaviors of others. |
| 1. Need | The degree to which opinion leaders have deficits related to survival, well-being, or personal fulfillment, which will be addressed by implementation and/or delivery of the innovation. |
| 2. Capability | The degree to which opinion leaders have interpersonal competence, knowledge, and skills to fulfill Role. |
| 3. Opportunity | The degree to which opinion leaders have availability, scope, and power to fulfill Role. |
| 4. Motivation | The degree to which opinion leaders are committed to fulfilling Role. |
| D. Implementation Facilitators: Referrers, i.e., healthcare professionals | Individuals with subject matter expertise who assist, coach, or support implementation. |
| 1. Need | The degree to which implementation facilitators have deficits related to survival, well-being, or personal fulfillment, which will be addressed by implementation and/or delivery of the innovation. |
| 2. Capability | The degree to which implementation facilitators have interpersonal competence, knowledge, and skills to fulfill Role. |
| 3. Opportunity | The degree to which implementation facilitators have availability, scope, and power to fulfill Role. |
| 4. Motivation | The degree to which implementation facilitators are committed to fulfilling Role. |
| E. Implementation Leads: Healthcare insurance companies | Individuals who lead efforts to implement the innovation. |
| 1. Need | The degree to which implementation leads have deficits related to survival, well-being, or personal fulfillment, which will be addressed by implementation and/or delivery of the innovation. |
| 2. Capability | The degree to which implementation leads have interpersonal competence, knowledge, and skills to fulfill Role. |
| 3. Opportunity | The degree to which implementation leads have availability, scope, and power to fulfill Role. |
| 4. Motivation | The degree to which implementation leads are committed to fulfilling Role. |
| F. Implementation Team Members | Individuals who collaborate with and support the Implementation Leads to implement the innovation, ideally including Innovation Deliverers and Recipients. |
| 1. Need | The degree to which implementation team members have deficits related to survival, well-being, or personal fulfillment, which will be addressed by implementation and/or delivery of the innovation. |
| 2. Capability | The degree to which implementation team members have interpersonal competence, knowledge, and skills to fulfill Role. |
| 3. Opportunity | The degree to which implementation team members have availability, scope, and power to fulfill Role. |
| 4. Motivation | The degree to which implementation team members are committed to fulfilling Role. |
| G. Other Implementation Support | Individuals who support the Implementation Leads and/or Implementation Team Members to implement the innovation. |
| 1. Need | The degree to which other implementation support have deficits related to survival, well-being, or personal fulfillment, which will be addressed by implementation and/or delivery of the innovation. |
| 2. Capability | The degree to which other implementation support have interpersonal competence, knowledge, and skills to fulfill Role. |
| 3. Opportunity | The degree to which other implementation support have availability, scope, and power to fulfill Role. |
| 4. Motivation | The degree to which other implementation support are committed to fulfilling Role. |
| H. Innovation Deliverers: Coaches | Individuals who are directly or indirectly delivering the innovation. |
| 1. Need | The degree to which innovation deliverers have deficits related to survival, well-being, or personal fulfillment, which will be addressed by implementation and/or delivery of the innovation. |
| 2. Capability | The degree to which innovation deliverers have interpersonal competence, knowledge, and skills to fulfill Role. |
| 3. Opportunity | The degree to which innovation deliverers have availability, scope, and power to fulfill Role. |
| 4. Motivation | The degree to which innovation deliverers are committed to fulfilling Role. |
| I. Innovation Recipients | Individuals who are directly or indirectly receiving the innovation. |
| 1. Need | The degree to which innovation recipients have deficits related to survival, well-being, or personal fulfillment, which will be addressed by implementation and/or delivery of the innovation. |
| 2. Capability | The degree to which innovation recipients have interpersonal competence, knowledge, and skills to fulfill Role. |
| 3. Opportunity | The degree to which innovation recipients have availability, scope, and power to fulfill Role. |
| 4. Motivation | The degree to which innovation recipients are committed to fulfilling Role. |
| **V. IMPLEMENTATION PROCESS DOMAIN** | **Implementation Process: The activities and strategies used to implement financial incentives.** |
| A. Teaming | The degree to which individuals join together, intentionally coordinating and collaborating on interdependent tasks, to implement the innovation. |
| B. Assessing Needs | The degree to which individuals collect information about priorities, preferences, and needs of people. Note: Use this construct to capture themes related to Assessing Needs that are not included in the subconstructs below. |
| 1. Innovation Deliverers | The degree to which individuals collect information about the priorities, preferences, and needs of deliverers to guide implementation and delivery of the innovation. |
| 2. Innovation Recipients | The degree to which individuals collect information about the priorities, preferences, and needs of recipients to guide implementation and delivery of the innovation. |
| C. Assessing Context | The degree to which individuals collect information to identify and appraise barriers and facilitators to implementation and delivery of the innovation. |
| D. Planning | The degree to which individuals identify roles and responsibilities, outline specific steps and milestones, and define goals and measures for implementation success in advance. |
| E. Tailoring Strategies | The degree to which individuals choose and operationalize implementation strategies to address barriers, leverage facilitators, and fit context. |
| F. Engaging | The degree to which individuals attract and encourage participation in implementation and/or the innovation. Note: Use this construct to capture themes related to Engaging that are not included in the subconstructs below. |
| 1. Engaging Innovation Deliverers (coaches) | The degree to which individuals attract and encourage deliverers to serve on the implementation team and/or to deliver the innovation. |
| 2. Engaging Innovation Recipients | The degree to which individuals attract and encourage recipients to serve on the implementation team and/or participate in the innovation. |
| 3. Engaging Referrers | The degree to which individuals attract and encourage referrers to serve on the implementation team and/or participate in the innovation. |
| G. Doing (pilots) | The degree to which individuals implement in small steps, tests, or cycles of change to trial and cumulatively optimize delivery of the innovation. |
| H. Reflecting & Evaluating | The degree to which individuals collect and discuss quantitative and qualitative information about the success of implementation. Note: Use this construct to capture themes related to Reflecting & Evaluating that are not included in the subconstructs below. |
| 1. Implementation | The degree to which individuals collect and discuss quantitative and qualitive information about the success of implementation. |
| 2. Innovation | The degree to which individuals collect and discuss quantitative and qualitative information about the success of the innovation. |
| I. Adapting | The degree to which individuals modify the innovation and/or the Inner Setting for optimal fit and integration into work processes. |
| J. Communicating (+) | The communication strategies adopted. |
| 1. Communication strategy society (+) | The communication strategies targeting society. |
| 2. Communication strategy recipients (+) | The communication strategies targeting recipients. |
| 3. Communication strategy politicians (+) | The communication strategies targeting politicians. |
| 4. Communication strategy insurers (+) | The communication strategies targeting insurers. |
| 5. Communication strategy policy makers (+) | The communication strategies targeting policymakers. |
| 6. Communication strategy referrers (+) | The communication strategies targeting referrers. |

*Codebook used for thematic analysis, based on the Consolidated Framework for Implementation Research (CFIR). The inner setting and outer setting domains were merged into a single domain, implementation setting, to reflect the interdependence between delivery organizations and external financing and policy structures in the Dutch smoking cessation care context. Codes added inductively are marked with (+)*
