# Supplementary material for: Stakeholder Perspectives on National Implementation of Financial Incentives for Smoking Cessation Before, During and After Pregnancy: A Policy Lab Study
Source: Health Expect. 2026 Oct 12;29(5):e70893. doi: 10.1111/hex.70893 (PMC13656341; doi:10.1111/hex.70893)
Supplement: Supplementary file 6 [file HEX-29-e70893-s005.docx]

#### Supplementary File 6: Overview of implementation factors

| Code | # | Example quote |
| --- | --- | --- |
| I. INNOVATION DOMAIN |  |  |
| Insight and fidelity in cost-effectiveness | 17 | "That it’s placed with the health insurers to finance this, but that they’re not sufficiently convinced of the cost-effectiveness (...) Because the cost-effectiveness for them, from their perspective, is mostly in the long term, and not cost-effective enough to invest in now. And it could also happen that one insurer invests money, but the benefits end up with another insurer." |
| Form of incentive | 17 | "No cash reward, but a discount on the (supplementary) health insurance to improve feasibility and acceptability (fairness), or rewards in the form of products/services for the child." |
| Evidence-Base | 16 | "Cost-effectiveness compared to the current standard practice has been demonstrated." |
| Relative Advantage | 15 | "But it also needs to be made very clear that the cost-effectiveness is extremely high, so that the Dutch society as a whole benefits. That’s important for politics, to ultimately build public support." |
| Selective targeting | 13 | "Offering the same incentive/reward to non-pregnant smokers makes it more equal (though possibly less cost-effective)." |
| Complexity | 12 | "The infrastructure, that’s still quite complicated when it comes to rewards, and could become a bottleneck. A coach would have to offer the rewards, and there needs to be biochemical validation to check whether someone has quit. That requires a whole infrastructure, so the further organisation of this could be a barrier." |
| Gaming and biochemical validation | 12 | "Breath tests could be performed close to home, in places people visit regularly (GP, midwife, child health centre, etc.)." |
| Innovation Cost | 11 | "Introduce an annual cap on expenses if costs risk becoming too high > participation possible again the following year." |
| Coaching program design and quality | 9 | "Embed incentives within a strong intervention including pharmacological and/or psychosocial support." |
| Innovation Design | 5 | "What I find elegant about incentives is that you don’t always need to explain them in detail, and that they could provide support through a non-cognitive route. At the same time, it might also be valuable not to put too much emphasis on the incentive aspect within the broader intervention. It’s simply a complementary element within a broader approach that we know is more effective and leads to better outcomes of the same programme." |
| Trialability | 5 | "You just make sure to test it among the group of people insured with that company." "Yes. And within Smoke-free Parents, something like that could be arranged, that providers who have a contract with a specific insurer can also offer the reward." |
| Integration in existing program | 3 | "That’s where it becomes more complicated to implement, if you have to do it at several time points." "Yes, but coaching also takes place at several time points, right? I think you just need to give an example of what such a programme could look like." |
| Innovation Source | 3 | "People didn’t want to participate because they were afraid the health insurer would use their data." |
| Adaptability | 1 | "I can also imagine doing something like vouchers, so that parents can choose what they actually need most at that moment." |
| II. SETTING DOMAIN |  |  |
| Financing | 36 | "The willingness and ability to finance prevention and rewards could be a barrier. So whether the government wants to fund prevention, whether health insurers are willing, but also whether they’re able to, and whether it’s legally and politically feasible." |
| Local Attitudes | 34 | "[Risk] The public considered this unfair (because they quit smoking without receiving a reward)." |
| Policies & Laws | 24 | "Legally, you could say that a judge might disagree with creating inequality, if some people receive a reward and others don’t, and could potentially intervene." |
| External Pressure | 13 | "There could be a lot of public commotions on social media: ‘It’s ridiculous that these people get help while those who behave well get nothing. That’s unfair.’ And then the political pressure could lead to stopping the programme." |
| Financing from insurers’ own budgets or supplementary insurance | 11 | "We have also invested in lifestyle hubs and such. That’s also prevention, and the benefits also come only in the long term. Apparently, they found that interesting enough to do, so it’s not that insurers are completely unwilling." |
| Structural Characteristics | 10 | "Logistical problems for distributing the rewards." |
| Basic insurance | 8 | "It’s not possible to give rewards from an individual health insurance plan." |
| Partnerships & Connections | 5 | "The general implementation of the programme runs through the *Smoke-free Start* project (Trimbos Institute), to ensure that both healthcare providers and the target group are aware of it." |
| Compatibility | 5 | "That’s where it becomes more complicated to implement, if you have to do it at several time points.” “Yes, but coaching also takes place at several time points, right? I think you just need to give an example of what such a programme could look like.” |
| Tension for Change | 4 | "To make them aware of their responsibility. Like: so many children and mothers suffer from this, and if you can make that clear with numbers, they must act when such a promising intervention exists." |
| Local Conditions | 4 | "In politics, you might think they don’t have or don’t want to allocate money at the moment to also give rewards. There are current budget cuts and so on." |
| Culture | 3 | "I’m not jealous that someone gets maternity care, for example. I completely agree. We should be in solidarity with each other when it comes to prevention, just as we are with curative care." |
| Incentive Systems | 3 | "That it’s convenient for their insured clients and attractive for recruiting people, that’s also a marketing consideration, of course." |
| Mission Alignment | 3 | "Didn’t they also sign the National Prevention Agreement? If they’re part of that, then you can hold them accountable for it, right?" |
| Relational Connections | 2 | "Should *Zorgverzekeraars Nederland* (the association of health insurers) also be involved, since they could ensure collective action among insurers?" |
| Communications | 2 | "This is really a topic the media are very interested in. I’ve noticed that before. Whenever we publish something about smoking and reach out to the media, they respond immediately. And *participant* has also been on television several times. So you can also use that proactively." |
| Relative Priority | 2 | "Focus on building political support: 'they need to invest in it and take a stand, because it’s very important'. And how do we do that? By clearly presenting the problem and appealing a bit to emotions. Like: we’re cutting education budgets because healthcare costs are rising, but those costs are increasing because so many people smoke, as shown again in the latest research." |
| Access to Knowledge & Information | 2 | "In addition to training for healthcare providers, so that they are really informed about this. Because it simply doesn’t work if providers are unaware." |
| Critical Incidents | 2 | "[Risk] Media have reported cases of misuse or fraud." |
| Available Resources | 1 | "Too many parents participated (capacity/budget issue)." |
| III. INDIVIDUALS DOMAIN |  |  |
| Innovation Recipients | 32 | "Programme is complex: how does it work? If you target low SES groups, you need to be super clear. Provide support to get started." |
| Politicians | 23 | "Perhaps something [a reward] specifically for the future child, which also encourages a healthier lifestyle, like a sports membership or a play area subscription." "Well, I think that might also be more acceptable for politicians." |
| Healthcare insurance companies | 15 | "Why it’s really important to clearly explain the financial aspect is because, for most people, the idea of paying people to quit smoking is just so strange. It feels absurd to politicians, to health insurers, on an emotional level, you know…" |
| Referrers | 10 | "Many healthcare providers, midwives, don’t even know that this exists. So if they don’t know about it, they can’t offer it." |
| Coaches | 6 | "Engaged smoking cessation providers are reluctant to take on additional tasks." |
| Policymakers | 2 | "'Smoking is a serious addiction', that’s true, of course, but if you start with that, many people will probably say: 'Yes, but people should just be strong enough themselves.'" "I don’t think policymakers see it that way. (...) More like: 'It’s their own choice.' (...) They’re in this setting, so it’s not new to them." |
| IV. IMPLEMENTATION PROCESS DOMAIN |  |  |
| Communication strategy society | 23 | "I think you should frame it as treatment for a disease: smoking is an addiction, and you want the treatment that works best, which is coaching with incentives." |
| Communication strategy recipients | 13 | "National campaign on TV, leaflets at daycare centres, schools, midwifery practices, hospitals, indoor playgrounds, GP practices, and baby gift boxes, etc." |
| Engaging Innovation Recipients | 13 | "I would involve the target group in the thinking process, especially regarding the video. You could ask several people what they think politicians should know. There’s quite a bit of criticism that it’s paternalistic. You can counter that by showing that the target group actually wants this too." |
| Communication strategy politicians | 10 | "So definitely contact the Ministry of Health, because they have the Prevention Agreement and similar initiatives. If you can link it to that and use their influence to get insurers on board…" |
| Doing (pilots) | 9 | "A successful pilot helps a lot, so that the health insurer can present it to Health Insurers Netherlands and then persuade others about it." |
| Communication strategy insurers | 8 | "What amount are we talking about per insurer? Large or small insurers... I think it’s really important to have a clear cost overview of what it might cost and then also present the benefits clearly." |
| Engaging Referrers | 6 | "Involve multiple parties who are in contact with low-SES smokers, such as general practitioners, debt counsellors, midwives, and social workers." |
| Communication strategy policy makers | 5 | "And if the insurers, but also the government, immediately ask 'how much does it cost?' that should be ready, a proposal." "So I think that financial picture needs to be clearer." |
| Tailoring Strategies | 5 | "When you focus more on local institutions such as community centres or child centres, and they have the right information, they can immediately connect with the target group. They often already have someone who speaks their language and can help them sign up, and so on." |
| Teaming | 4 | "You could of course involve all sorts of organisations, like the Lung Foundation, or others. If you invite them to help spread the word, that really helps. People will start talking about it, and that in turn helps policymakers and health insurers, I think." |
| Assessing Needs | 4 | "Clear agreements with involved stakeholders, identifying what is needed and who fulfils which role." |
| Communicating | 4 | "Communicate that smoking is a disease, share the (cost-)effectiveness of incentives on (social) media, and share the costs of smoking." |
| Engaging | 3 | "Proper information provision, right? Make sure it reaches the right people. Also when it comes to lobbying towards politics: what are their interests? Include that in your message." |
| Assessing Context | 2 | "Engage in discussions with the Ministry of Health, Health Insurers Netherlands, and/or the National Health Care Institute about the barriers and necessary conditions for financing incentives." |
| Planning | 2 | "I think you all need to decide together what the most promising route is, and then adapt the policy brief accordingly, as well as the way you distribute it." |
| Communication strategy referrers | 2 | "Involve multiple parties who are in contact with low-SES smokers, such as GPs, debt counsellors, midwives, and social workers. Broad information dissemination." |
| Engaging Innovation Deliverers | 1 | "Ensure there is sufficient support from coaches." |

*Overview of identified barriers and facilitators to implementation, organized by CFIR domain (intervention characteristics, implementation setting, individual actors, and implementation process). The table presents codes, their frequency of occurrence (#), and illustrative example quotes.*
